# Biological Features of KLC2 Mutations in Chronic Myeloid Leukemia and Their Contribution to Inducing Drug Resistance

**DOI:** 10.32604/or.2025.070259

**Published:** 2025-12-30

**Authors:** Rabindranath Bera, Yotaro Ochi, Ying-Jung Huang, Ming-Chung Kuo, Kenichi Yoshida, Seishi Ogawa, Lee-Yung Shih

**Affiliations:** 1 Division of Hematology-Oncology, Chang Gung Memorial Hospital-Linkou, Taoyuan City, 333, Taiwan; 2 Department of Pathology and Tumor Biology, Graduate School of Medicine, Kyoto University, Kyoto, 6068501, Japan; 3 Department of Hematology and Oncology, Graduate School of Medicine, Kyoto University, Kyoto, 6068501, Japan; 4 School of Medicine, Chang Gung University, Taoyuan City, 333, Taiwan; 5 Division of Cancer Evolution, National Cancer Center Research Institute, Tokyo, 1040045, Japan

**Keywords:** Chronic myeloid leukemia (CML), kinesin light chain 2 (KLC2), signal transducer and activator of transcription 3 (STAT3), drug resistance, myeloid blast transformation

## Abstract

**Background:**

Breakpoint Cluster Region-Abelson (BCR::ABL1) fusion protein is essential in the pathogenesis of chronic myeloid leukemia (CML); however, the chronic-to-blast phase transformation remains elusive. We identified novel kinesin light chain 2 (*KLC2*) mutations in CML-myeloid blast phase patients. We aimed to examine the functional role of *KLC2* mutations in leukemogenesis.

**Methods:**

To evaluate the biological role of KLC2 mutants (MT) in CML cells, we expressed *KLC2-MT* in different human CML cell lines harboring *BCR::ABL1* and performed immunoblot, immunofluorescence, cell proliferation, differentiation, and apoptosis; Tyrosine kinase inhibitor (TKI)-drug activities; and clonogenic assays for *in vitro* functional analyses. We co-expressed *KLC2-MT* and *BCR::ABL1* in mouse bone marrow cells (BMCs) to evaluate their clonogenic and self-renewal abilities *ex vivo*. Furthermore, we examined tumorigenic activity and drug efficacy in the K562 xenograft model.

**Results:**

*KLC2-MT* overexpression in *BCR::ABL1-*positive K562 and KU812 CML cells promoted cell proliferation and clonogenic potential, decreased imatinib sensitivity, and reduced apoptosis. Serial colony replating assays revealed that KLC2-MT and BCR::ABL1 co-expression enhanced the self-renewal ability of mouse BMCs with immature morphology. In the K562 xenograft model, KLC2-MT enhanced tumorigenic potential and diminished imatinib efficacy. Further studies reported that KLC2-MT augmented signal transducer and activator of transcription 3 (STAT3) activation and nuclear accumulation in imatinib-treated CML cells. KLC2-WT and KLC2-MT interacted with mothers against decapentaplegic homolog 2 (SMAD2); however, the latter impaired transforming growth factor-beta (TGF-β)–mediated SMAD2/3 activation while enhancing STAT3 phosphorylation.

**Conclusions:**

This study demonstrates the biological and functional importance of KLC2 mutation in CML cells, potentially enabling the development of better treatment strategies for CML patients carrying *KLC2* mutations and providing enhanced understanding of the disease progression.

## 1 Introduction

The acquisition of the oncoprotein Breakpoint Cluster Region-Abelson (BCR::ABL1), a constitutively active tyrosine kinase, induces leukemogenesis of chronic myeloid leukemia (CML), which progresses from the chronic phase (CP) to the accelerated phase and ultimately to the terminal blast phase (BP), resulting in an unfavorable outcome [1–3]. Tyrosine kinase inhibitors (TKIs) or allogeneic stem cell transplantation can inhibit or slow down the progression of CML; however, some CML patients develop resistance to TKIs, particularly those who progress to advanced stages of the disease [1,4,5]. Imatinib mesylate does not completely eradicate CML in some patients, as evidenced by primary resistance [6–8]. Mutations in the BCR::ABL1 kinase domain are among the leading causes of secondary resistance to TKIs and risk factors for blastic transformation [9–11]. Blastic transformation requires the acquisition of additional genetic abnormalities; however, the molecular mechanisms of mutation acquisition and resistance to TKIs are not completely understood. CML progression to myeloid BP is potentially caused by the acquisition of additional driver mutations. Therefore, understanding the mechanism of additional driver mutations or pathways acquired during blastic transformation is crucial.

We recently performed whole-exome sequencing on 52 matched-pairs paired samples at diagnosis and at BP of CML, as well as targeted capture sequencing in 60 CML-BP samples; then, we determined the genetic changes that contributed to CML-BP clonal evolution [12]. Furthermore, we identified recurrent novel kinesin light chain 2 (*KLC2*) gene mutations in CML-BP patients and other myeloid malignancy–related genes, including *RUNX1*, *ABL1*, *ASXL1*, *BCOR/BCORL1*, *TP53*, and *WT1* [12]. The *KLC2* gene is involved in intracellular transport in different cell types [13–15]. The aberration of this gene resulted in neuropathy syndrome, ocular atrophy, and spastic paraplegia [16]. KLC2 regulates Lemur tyrosine kinase-2, a prostate cancer susceptibility gene, and transforming growth factor-β–induced Smad2 signaling [17,18]. However, the biological roles and contribution of *KLC2* mutations in CML-to-CML-BP transformation have not yet been investigated. We hypothesized that the acquisition of *KLC2* mutation during CML-CP-to-myeloid BP contributes to disease progression.

We conducted this study to assess the functional significance of KLC2 mutations, which could help improve understanding of the disease and lead to more effective treatments for a subgroup of CML patients, as CML-CP progresses to myeloid BP.

## 2 Materials and Methods

### 2.1 Ethical Approval

The ethics committee of the Chang Gung Memorial Hospital at Linkou approved the study. This approval was issued under decision number 201702163B0 on 02 January 2018. Each patient provided signed informed consent. All animal experiments were conducted at the Department of Animal Experimentation at Chang Gung Memorial Hospital (CGMH) at Linkou, approved by the Institutional committee (Approval number: 2017121824).

### 2.2 Patient Samples and Mutational Analysis

Details on bone marrow (BM) sample collection and mutational analysis were previously presented [12]. In brief, we performed whole-exome sequencing (WES), targeted capture sequencing, and/or deep amplicon sequencing, using 112 CML-BC and 71 CP samples at diagnosis from 130 patients. Combined with external WES data of 24 BC and 77 CP patients, we comprehensively analyzed a total of 136 BC and 148 CP samples from 216 CML patients for single-nucleotide variants (SNVs). Peripheral blood or bone marrow samples, along with matched buccal samples when available, were collected from enrolled patients. Genomic DNA was extracted using the QIAamp DNA Mini Kit (Cat#51104, Qiagen, Hilden, Germany) or Genetra PureGene Kit (Cat#158467, Qiagen).

To identify additional *KLC2* mutations, we did not filter mutations by variant allele frequency threshold. The Catalogue of Somatic Mutations in Cancer (COSMIC) database (https://cancer.sanger.ac.uk/cosmic, accessed on April 2022) was searched for mutations.

### 2.3 Cell Culture, Plasmid Construction, Lentiviral Preparation, and Infection

K562, KU812, U937, HL60, and HEL human leukemia cells were cultured in RPMI-1640 medium, and HEK293T cells were cultured in DMEM under standard conditions. RPMI-1640, DMEM, FBS, and antibiotic–antimitotic solution were purchased from Thermo Fisher Scientific (Waltham, MA, USA). K562, HL60, U937, and HEK293T cells were sourced from ATCC, and KU812 and HEL cells from Bioresource Collection and Research Center, Hsinchu, Taiwan. All cell lines used in this study were free from mycoplasma contamination. Cellular morphology and short tandem repeat analysis were employed to confirm the authentication of K562, KU812, and U937 leukemia cell lines at Genomics, BioSci. & Tech. Co., Ltd., New Taipei city, Taiwan (2022). We followed our previous description for plasmid construction as well as lentiviral production and infection [19]. The procedures are presented in the Supplementary Methods. Knocked down *KLC2* from K562 and KU812 cells, and STAT3 from K562 cells transduced with KLC2-WT and MT was described in the Supplementary methods.

### 2.4 Drug Treatment, Staining, Cell Proliferation, and Colony-Forming Assays

Cell viability was evaluated either manually using a hemocytometer (DIN 12847, Blankenburg, Germany) and the trypan blue exclusion assay or using CCK-8 reagents (Catalog No. TEN-CCK8, Biotools Co., Ltd., Taipei, Taiwan) according to the manufacturer’s protocols. For the trypan blue exclusion assay, the diluted cell suspension was mixed with the trypan blue dye (1:1 ratio), and counted viable cells (bright) excluding non-viable cells (blue), using a hemocytometer under a microscope. The parental and transformed K562 and KU812 cells were cultured in the presence of imatinib (Novartis, Basel, Switzerland) (0.05 to 10 μm) and dasatinib (Pharmascience Inc., Montreal, QC, Canada) (0.05 to 10 nm) at varying concentrations or under drug-free conditions for 72 h. IC_50_ values of these drugs for parental and transformed K562 and KU812 cells were calculated. KLC2-WT/MT-expressing K562 and KU812 cells were subjected to treatment with 400 and 100 nM imatinib, respectively, or untreated for 72 h for assessments of cell viability and apoptosis; similar K562 and KU812 cells were administered 0.4 nM dasatinib or remained drug-free for 72 h for analyses of cell viability. To analyze the expression of cell signaling proteins, similar transformed K562 and KU812 cells were treated with 1 μm imatinib and 1 nM dasatinib or untreated for 20 h. K562-WT/MT-expressing cells were starved overnight before receiving 10 ng/mL TGF-β for one hour in order to test the impact of TGF-β. To evaluate the stability of pSTAT3, stable KLC2-WT/MT-expressing K562 cells and transiently-expressing 293T cells were treated with 1 and 10 μm imatinib for 20 h, followed by the addition of cycloheximide (CHX) at a concentration of 100 μg/mL for 6 h. Only dimethyl sulfoxide was used to treat the control cells. Clonogenic growth assays and morphological studies were conducted, as previously described [20]. For clonogenic growth assays, transformed stable KLC2-WT/MT-expressing K562, KU812, and U937 and EV control cells were cultured in 12-well plate at 1–2 × 10^3^ cells/well in 1% methylcellulose containing RPMI medium supplemented with 10% FBS for 10 days. Photograph was taken by phase contrast microscope (Nikon Eclipse TS100, Kawasaki, Japan). For morphological studies, cytospined smears were stained with Liu’s reagents (Jen An Technology Co., Ltd., Kaohsiung, Taiwan). Digital images were acquired using Olympus (model no. U-TV0.5XC-3, Tokyo, Japan) microscope equipped with a digital camera.

### 2.5 Protein Extraction, Co-Immunoprecipitation (Co-IP), and Immunoblotting

Cell lysate preparation and Western blotting were performed as previously described [21]. Briefly, KLC2-WT/MT-expressing U937, K562, and KU812 cells treated with or without drugs were homogenized and lysed in a buffer containing 50 mM Tris-HCl, pH 8.0, 120 mM NaCl, 0.5% NP-40, 0.25% Na deoxycholate, 1 mM DTT, 1 mM EDTA, 1 mM NAF, 1 mM Na3VO4, and protease inhibitor (all chemicals purchased from Sigma Chemicals, St. Louis, MO, USA). A total of 20–40 μg proteins were subjected to electrophoresis followed by immunoblotting analysis using antibodies against the target proteins. A loading control, either of detection of β-Actin or GAPDH, was included for all immunoblots. The protein lysate was resolved in 6%–12% SDS polyacrylamide gels according to the protein size, transferred to a nylon membrane (PVDF, Schleiche & Schuell, Einbeck, Germany), and then incubated with primary antibodies at 4°C overnight. After washing, the membranes were incubated with HRP-conjugated anti-mouse IgG or anti-rabbit antibodies for 1 h. Detection was completed using enhanced chemiluminescence (ParkinElmer, Shelton, CT, USA). Nucleocytoplasmic protein was isolated using a cytoplasmic and nuclear protein extraction kit (Catalog No. BRARZ106 Biotools Co., Ltd., Taipei, Taiwan) according to the manufacturer’s protocols. The antibodies used and details of the dilutions are presented in Supplementary Table S1. FLAG-KLC2-WT, R312W, and L523I were transiently expressed in HEK293T cells, and co-IP was performed as previously described [22]. Briefly, FLAG KLC2-WT/MT-expressing HEK293T cells were washed with ice-cold PBS, pH 7.4 (Thermo Fisher Scientific, Waltham, MA, USA) twice and lysed in lysis buffer (150 mM NaCl, 50 mMTrisHClpH7.4, 0.5%NP-40) supplemented with protease and phosphatase inhibitors. Lysates containing an equal amount of protein (500–1000 μg) were subjected to immunoprecipitation with anti-FLAG M2 affinity gel (Sigma-Aldrich, Cat. A2220, St. Louis, MO, USA) at 4°C overnight, according to the manufacturer’s instructions. The lysates were then washed and eluted by boiling in Laemmli buffer. Immunoblotting was analyzed as described above.

### 2.6 Immunofluorescence Analysis

Immunofluorescence studies were conducted as previously described [21]. Briefly, PBS, pH 7.4 (Thermo Fisher Scientific, Waltham, MA, USA)-washed cytospin KLC2-WT/MT-expressing K562 cells were fixed with chilled 100% methanol for five minutes at room temperature, washed thrice with cold PBS, blocked with 5% BSA in PBS containing 0.1% Tween 20 for 1 h, and then incubated with the primary antibodies at 4°C overnight. Following three rounds of washing, the cells were incubated with FITC-conjugated secondary antibodies for 1 h and counterstained with 4,6-diamidino-2-phenylindole (DAPI). Cells were washed and mounted, morphological changes in cells were examined under a fluorescence microscope (Olympus BX51 or BX61, Tokyo, Japan), and images were acquired with a digital color camera using either the Case Data Manager DP2-BSW (ver.2.1) or EXPO 6.0 software system (Olympus, Tokyo, Japan). All the images had fixed contrast, with an exposure time of 80 ms for DAPI and 1000 ms for green fluorescence.

### 2.7 Flow Cytometry Analysis

Flow cytometry analysis was conducted as previously described [22]. In brief, after being treated with 400 and 100 nM imatinib for 72 h, KLC2-WT/MT-expressing K562 and KU812 cells were collected, washed in PBS, and counted. For the purpose of analyzing apoptosis, 5 × 10^5^ cells were subsequently washed with PBS containing 1% bovine serum albumin. Apoptosis was detected via Annexin V antibody and Propidium iodide staining, according to the manufacturer’s protocols (Annexin V-FITC kit, #556547, BD Biosciences, San Jose, CA, USA). The percentage of apoptotic cells was determined using a BD Aria III (BD Biosciences, Milpitas, CA, USA) cytometer and investigated using the Diva software (ver.6.1.3, BD Biosciences, San Jose, CA, USA).

### 2.8 Mouse and Ex Vivo Bone Marrow Colony-Forming Assay

All animal experiments were approved by the Department of Animal Experimentation at Chang Gung Memorial Hospital (CGMH) at Linkou (approval number: 2017121824). *Ex vivo* experiments were conducted using bone marrow cells (BMCs) of C57BL/6 mice from NAR LABS, Taipei, Taiwan. One female mouse aged 8–12 weeks was utilized for BMC isolation each time. BMC isolation and culture, lentiviral transduction, colony-forming assays, self-renewal activity, and colony type determination were performed as previously described [22]. Briefly, the mouse was injected with 5-fluorouracil (150 mg/kg) 4 days prior to BM isolation. After red blood cell (RBC) removal using an RBC lysis buffer (Sigma-Aldrich, Cat#R7757), the BMCs were cultured with RPMI containing 20% FBS, 2 mM L-glutamine, 1× antibiotic–antimitotic solution, 100 ng/mL mouse stem cell factor, and 10 ng/mL mouse IL-3. After a 48 h incubation, nonadherent cells were collected and transduced with BCR::ABL1 and BCR::ABL1/KLC2-WT/KLC2-R312W/KLC2-L523I, including EV constructs using lentivirus-mediated gene transfer methods. For colony-forming and self-renewal activity assay, 2 × 10^4^ transduced BM cells were mixed with 2 mL MethoCult medium (MethoCult M3434; Stem Cell Technologies, Vancouver, BC, Canada) in duplicate cultures in a 6-well tissue-cultured plate. Colonies were scored on day 8 of culture. Serial replating assays were performed on day 8 of the previous culture. All cells were harvested, washed twice with RPMI medium, and counted. A similar number of cells was then replated, and the process was repeated four times to check colony formation and self-renewal activity.

### 2.9 Tumorigenicity in Nude Mice

Female athymic nude mice (BALB/c-nu, 4- to 6-week-old) were purchased from NAR LABS, Taipei, Taiwan, for xenograft tumor formation experiments. A K562 cell xenograft tumor model was developed through subcutaneous inoculation of 7 × 10^6^ K562 cells with stable expression of KLC2-WT/MT or EV in BALB/C nude mice, utilizing 100 μL of RPMI-1640 medium mixed with 100 μL of Matrigel at two sites per mouse (3 and 4 mice in total per group, with and without drug treatment, respectively). After a 10-day inoculation, the mice were randomized to the control group (receiving the vehicle alone) or the drug-treated group and orally administered 100 mg/kg imatinib for 15 days (5 days/week) [23]. Tumor formation was observed, and the tumor size was measured using a caliper.

### 2.10 Statistical Analysis

GraphPad Prism 10 and Microsoft Excel 2016 were used for statistical analysis. Statistical analysis was conducted, as indicated in the figure captions. The significance of the differences between the groups was determined via ordinary one-way analysis of variance or unpaired *t*-tests. Data were expressed as means ± standard deviation (SD) from at least three independent experiments, unless otherwise specified. A *p*-value < 0.05 was considered significant for all analyses.

## 3 Results

### 3.1 KLC2 Mutations in CML and Human Cancers

**Figure 1:**
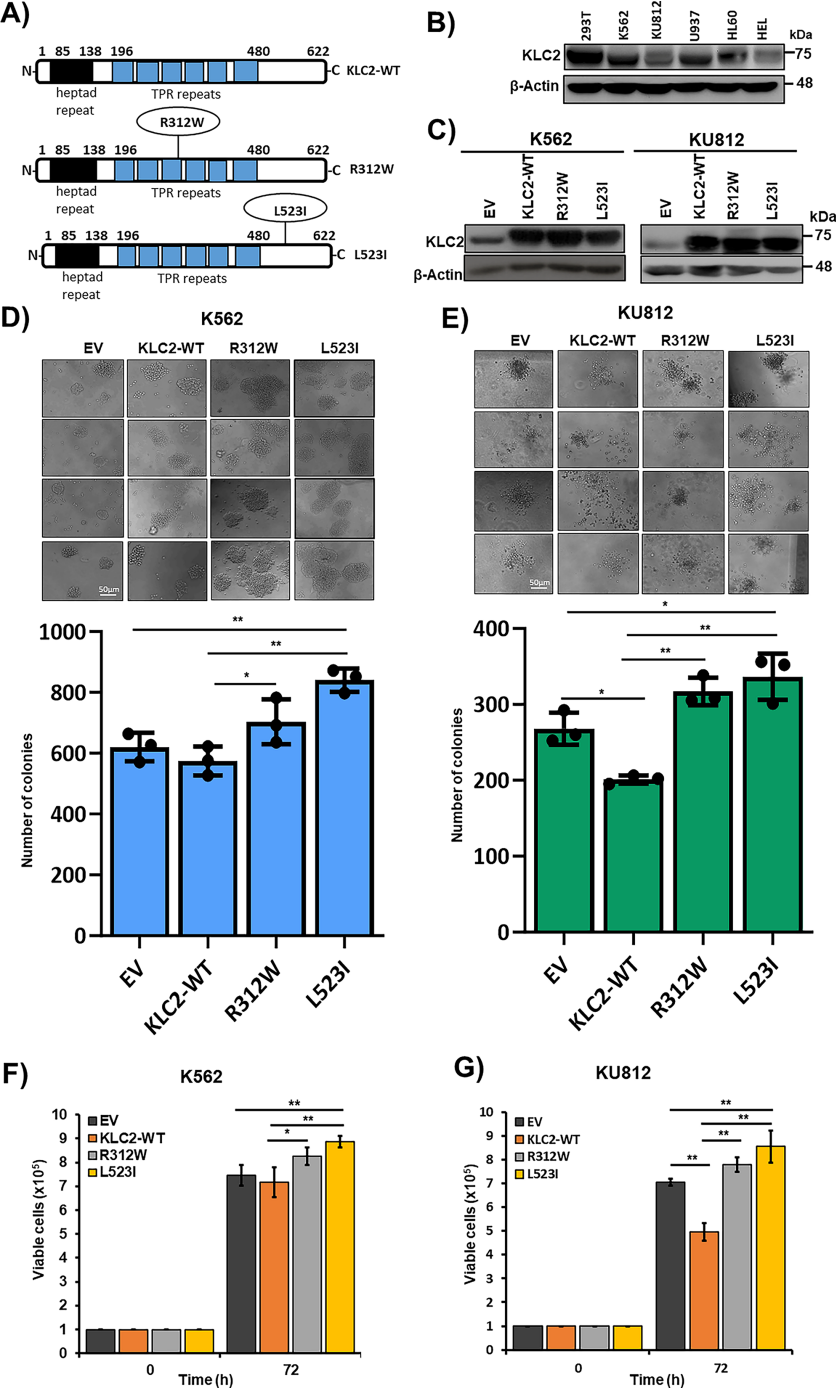
KLC2 mutants provide proliferation advantage and clonogenic potential in human CML cells. (**A**) Schematics of the full-length KLC2-WT, R312W and L523I mutants are shown. TPR, tetratricopeptide repeat domain. (**B**) Immunoblot of endogenous expression of KLC2 in different leukemia cell lines, including the HEK293T cell line. (**C**) KLC2-WT, R312W, and L523I expressions were checked in transformed K562, and KU812 by Immunoblotting. β-Actin was used as a control for equal loading. (**D**,**E**) Colony-forming assay was performed after the stable expression of KLC2-WT, R312W and L523I mutants in K562 (**D**) and KU812 (**E**) cells. Scale bar = 50 μm. Columns represent mean (sum of technical triplicates, mean of three independent experiments) ± SD, *p < 0.05, and **p < 0.01. (**F**,**G**) K562 and KU812 transformed cells were grown for 72 h and viable cells were counted by the trypan blue exclusion method. Error bars represent the mean ± SD of four independent experiments, *p < 0.05, and **p < 0.01

First, we searched the COSMIC database for *KLC2* mutations in human malignancies; a total of 170 *KLC2* mutations causing amino acid changes were registered (as of April 2022) [24,25]. In addition to the two previously reported variants [12], a *KLC2* R312W mutation with a low variant allele frequency (2.4%) was identified under less-stringent filtering criteria (Supplementary Table S2). In the COSMIC database, *KLC2* mutations were distributed across the entire protein in different human cancer types (Supplementary Fig. S1); however, aside from the R312W mutation, two other mutations were recurrently detected in C-terminal–proximal regions in our cohort. We selected the KLC2-R312W and L523I mutations located in the middle-and C-terminal–proximal regions of KLC2 proteins, respectively, for the functional analysis (Fig. 1A).

### 3.2 KLC2 Mutants Provide Proliferation Advantage and Clonogenic Potential in Human CML Cells

To elucidate the functional role of KLC2 in CML, we first determined the endogenous protein level in human myeloid cells with or without *BCR::ABL1*, including HEK293T, a nonhematopoietic cell line (Fig. 1B). All the tested cell lines expressed KLC2 at moderate to high levels. Next, we stably expressed *KLC2* wild type (WT), R312W, L523I, and empty vector (EV) control in human myeloid cells harboring *BCR::ABL1* (K562 and KU812) [26,27] and in *BCR::ABL1*-negative cells (U937) (Fig. 1C, Supplementary Fig. S2A). We found that cells carrying *KLC2-*R312W and *KLC2-*L523I mutations enhanced colony formation and increased cell survival compared with WT, whereas the R312W mutant exerted a modest effect when compared with EV (Fig. 1D–G, Supplementary Fig. S2B–D). KLC2-L523I-transduced cells generated considerably greater and denser colonies than WT cells. Moreover, immunoblotting experiments revealed that KLC2-L523I was highly distributed in the nucleus compared with WT and KLC2-R312W mutant in K562 and 293T cells (Supplementary Fig. S3A–D).

### 3.3 KLC2 Mutants Decreased Sensitivity to TKI and Reduced Imatinib-Induced Apoptosis in CML Cells

**Figure 2:**
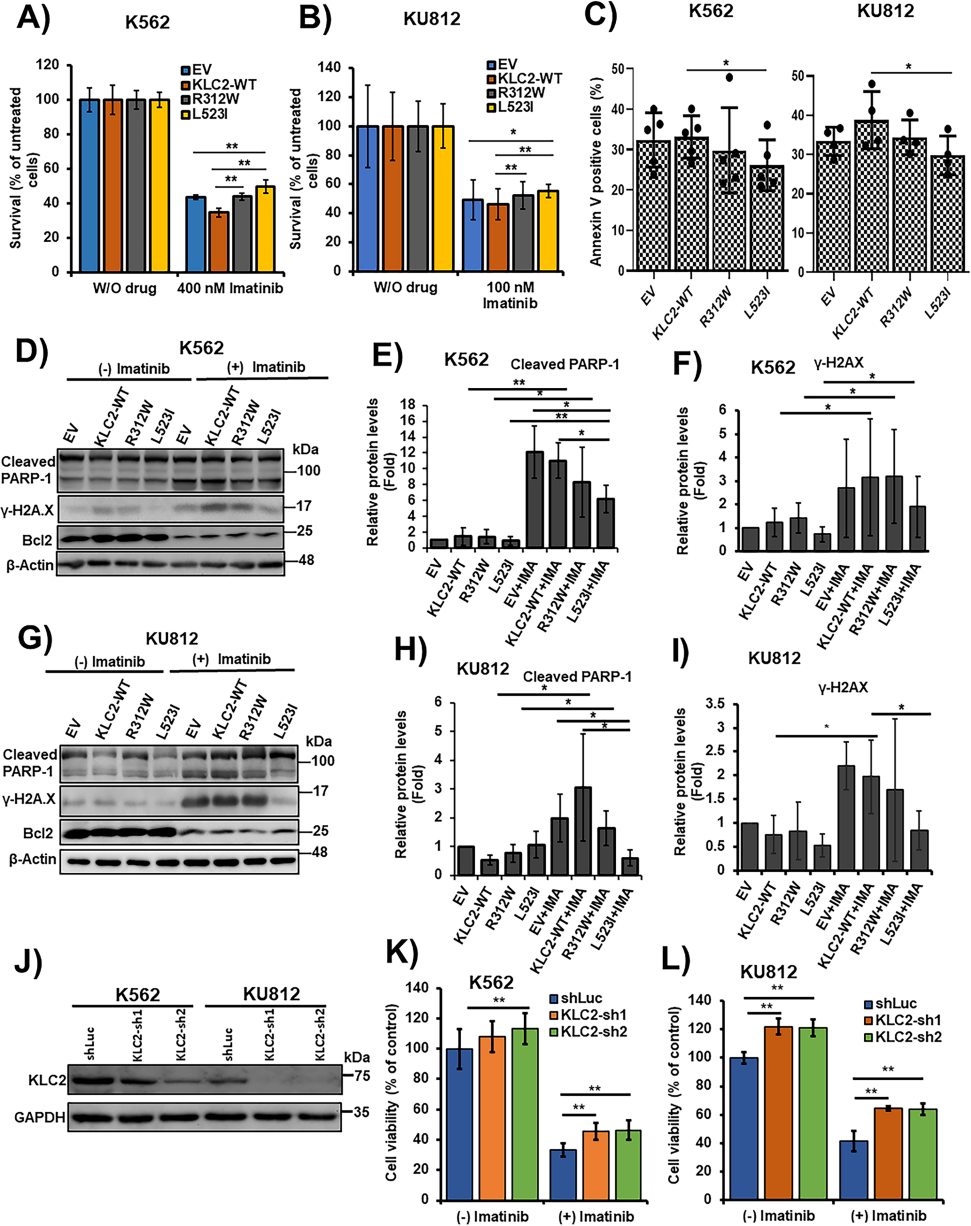
KLC2 mutants decreased the efficacy of CML cells to TKI and impaired imatinib-induced apoptosis. (**A**,**B**) KLC2-WT, R312W, and L523I mutants-expressing K562 cells were treated with 400 nM imatinib (**A**) and KU812 cells were treated with 100 nM imatinib (**B**) or without imatinib for 72 h. Drug-treated cells were normalized to untreated cells and the cell survival rate was evaluated using the trypan blue exclusion method. Error bars represent the mean ± SD of four independent experiments, *p < 0.05, **p < 0.01. (**C**) Similar K562 and KU812 cells were treated with 400 and 100 nM imatinib, respectively, for 72 h; the Annexin V positive cells (%) were analysed by flow cytometry. Error bars represent the mean ± SD of five (K562) and four (KU812) independent experiments, *p < 0.05. (**D**–**I**) Apoptosis-associated protein levels were examined with the treatment of Imatinib (400 nM) in K562 (**D**–**F**) and 100 nM in KU812 (**G**–**I**) cells for 72 h or without imatinib by immunoblotting, with β-actin being used for normalization. Non-treated EV cells were used as a control (**E**,**F**,**H**,**I**) for the calculation of fold change. Data were presented as the mean ± SD (n ≥ 3), *p < 0.05, **p < 0.01. (**J**) Stable knockdown of *KLC2* from K562 and KU812 cells and knockdown efficiency was checked by immunoblot analyses. (**K**,**L**) Knockdown of *KLC2* from K562 (**K**) and KU812 (**L**) cells were treated with or without 400 and 100 nM imatinib, respectively, for 72 h and viable cells were counted. Data were presented as a percentage of the viability of shLuc without imatinib treatment, considered as the control. Error bars represent the mean ± SD of five independent experiments; *p < 0.05, **p < 0.01. IMA indicated imatinib

K562 and KU812 cell viability was reduced by the inhibition of BCR::ABL1 tyrosine kinase activity by imatinib or dasatinib in a dose-dependent manner (Supplementary Fig. S4A–D). IC_50_ data indicated that KU812 cells were more responsive to drugs than K562 cells (Supplementary Fig. S4A–D). *KLC2*-WT, R312W, and L523I MTs stably expressing K562 and KU812 cells were cultured in the presence of imatinib and dasatinib. Both drugs effectively decreased cell survival at selective doses, with a smaller decrease observed in *KLC2-*R312W/L523I–expressing cells than in WT or control cells (Fig. 2A,B, Supplementary Fig. S5A,B). Moreover, the IC_50_ values indicated that either imatinib or dasatinib was less potent in *KLC2*-mutated cells compared with *KLC2*-WT cells (Supplementary Fig. S5C). However, the *KLC2*-L523I mutant was more resistant to the drugs than the R312W mutant. Similarly, flow cytometry analyses revealed that K562 and KU812 cells carrying *KLC2*-L523I expressed significantly lower Annexin V positivity than *KLC2*-WT in imatinib-treated cells (Fig. 2C). Immunoblot analyses showed that the increase of cleaved-PARP-1, γ-H2AX, and the decrease of BCL2 following imatinib treatment in transduced K562 or KU812 cells (Fig. 2D–I, Supplementary Fig. S5D,E), indicating the induction of apoptosis by imatinib. Contrarily, the expression levels of the indicated proteins differed between *KLC2*-mutated and *KLC2*-WT cells. Notably, *KLC2* knockdown from CML cells promoted cell growth, decreased imatinib sensitivity (Fig. 2J–L), and reduced cleaved PARP-1 indicating that the imatinib-induced apoptosis in CML cells required KLC2 (Supplementary Fig. S6A,B).

### 3.4 KLC2 Mutants Enhanced Tumorigenic Activity and Reduced the Sensitivity of CML Cells to Imatinib in Nude Mice

We developed a K562 cell xenograft model in nude mice via subcutaneous injection of K562 cells with a stable expression of *KLC2*-WT, R312W, L523I, or EV. We found that *KLC2*–R312W/L523I-transduced K562 cells demonstrated greater tumorigenic ability and higher tumor formation than *KLC2*-WT and EV control–transduced K562 cells (Supplementary Fig. S7A). The imatinib-treated and control groups were comparable in terms of tumor formation frequency (Supplementary Fig. S7A). Imatinib partially inhibited the growth of the transformed K562 xenograft. However, the suppression of tumor growth with imatinib was greater in *KLC2-*WT mice than in the control and L523I mice on the 15th day of the treatment, with reductions of 39% and 25% observed in mice carrying *KLC2*-WT and L523I, respectively (Supplementary Fig. S7B). Contrarily, imatinib-treated *KLC2*-R312W mice did not show growth suppression. We did not observe major differences in the weights of mice between the different experimental groups (Supplementary Fig. S7C).

### 3.5 KLC2 Mutants Enhanced STAT3 Activation and Nuclear Localization in Imatinib-Treated CML Cells

**Figure 3:**
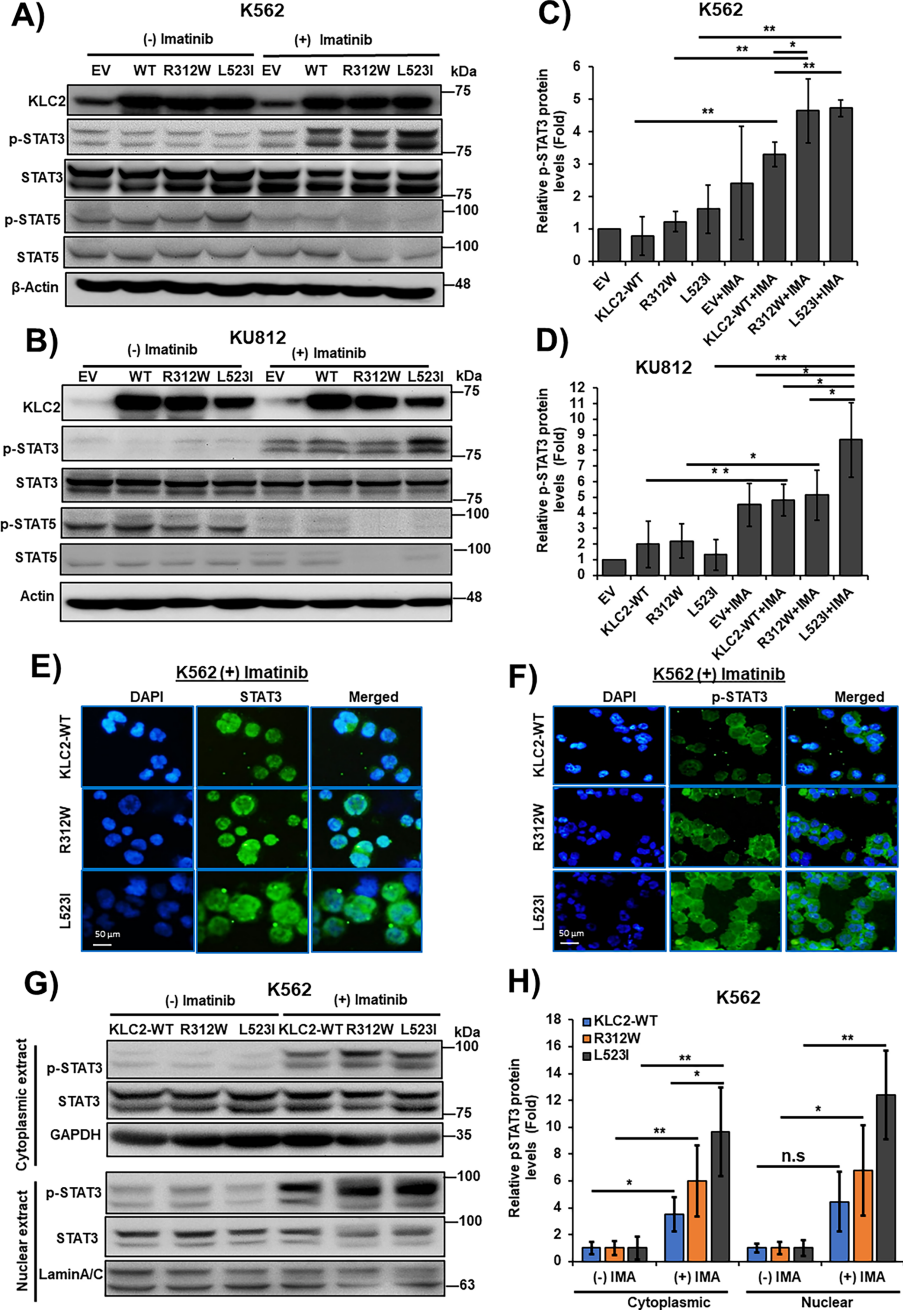
KLC2-mutants increased STAT3 activation and nuclear accumulation in Imatinib-treated CML cells. (**A**–**D**) KLC2-WT and R312W/L523I mutants-transduced K562 and KU812 cells were treated with 1 μm imatinib or without imatinib for 20 h and the indicated proteins were detected with specific antibodies. β-Actin was used for normalization. Non-treated EV cells were utilized as a control for the calculation of fold change (**C**,**D**). (**E**,**F**) 1 μm imatinib-treated K562 cells stably expressed KLC2-WT/MT for 20 h and the subcellular localization of the STAT3 (**E**) and p-STAT3 (**F**) proteins was visualized by immunofluorescence microscopy. Scale bar = 50 μm. Representative data are shown. (**G**,**H**) Cytoplasmic and nuclear proteins were extracted from similar K562 cells with or without imatinib treatment and the indicated proteins were detected by immunoblotting, normalized with GAPDH and Lamin A/C, respectively. Fold-change calculated with the comparison of non-treated cells. Error bars represent the mean ± SD (n ≥ 3), *p < 0.05, **p < 0.01. IMA, indicated imatinib; n.s, not significant

To elucidate the role of KLC2 MT on CML resistance to TKI, we examined a panel of cell proliferation signaling protein levels in *KLC2*-WT/R312W/L523I–transduced K562 and KU812 cells with and without imatinib and dasatinib treatments. We observed reduced ERK1/2, AKT, and STAT5 activation in the transduced K562 and KU812 cells following treatment with imatinib (Supplementary Fig. S8A,B, Fig. 3A,B) or dasatinib (Supplementary Fig. S9A,B). However, imatinib-and dasatinib-treated cells demonstrated enhanced STAT3 phosphorylation, which was greater in *KLC2*-mutated K562 and KU812 cells than in WT and EV cells (Fig. 3A–D and Supplementary Fig. S9A–D). Immunofluorescence and immunoblots results indicated STAT3 activation and nuclear accumulation in the presence of imatinib. The accumulation was higher in L523I cells than in *KLC2*-WT and R312W cells (Fig. 3E–H). Additionally, the activated STAT3 was more stable in *KLC2*–L523I mutant cells than in WT cells (Supplementary Fig. S10A–D). Next, we knocked down *STAT3* from K562 cells transduced with *KLC2-*WT and MT and found that *STAT3* knockdown reduced the proliferative advantage and imatinib resistance of transduced K562 cells (Supplementary Fig. S11A,B). Moreover, *KLC2* knockdown from K562 cells enhanced STAT3 phosphorylation (Supplementary Fig. S12A,B).

### 3.6 KLC2 Mutants Deregulated SMAD2/3 Signaling in CML Cells

**Figure 4:**
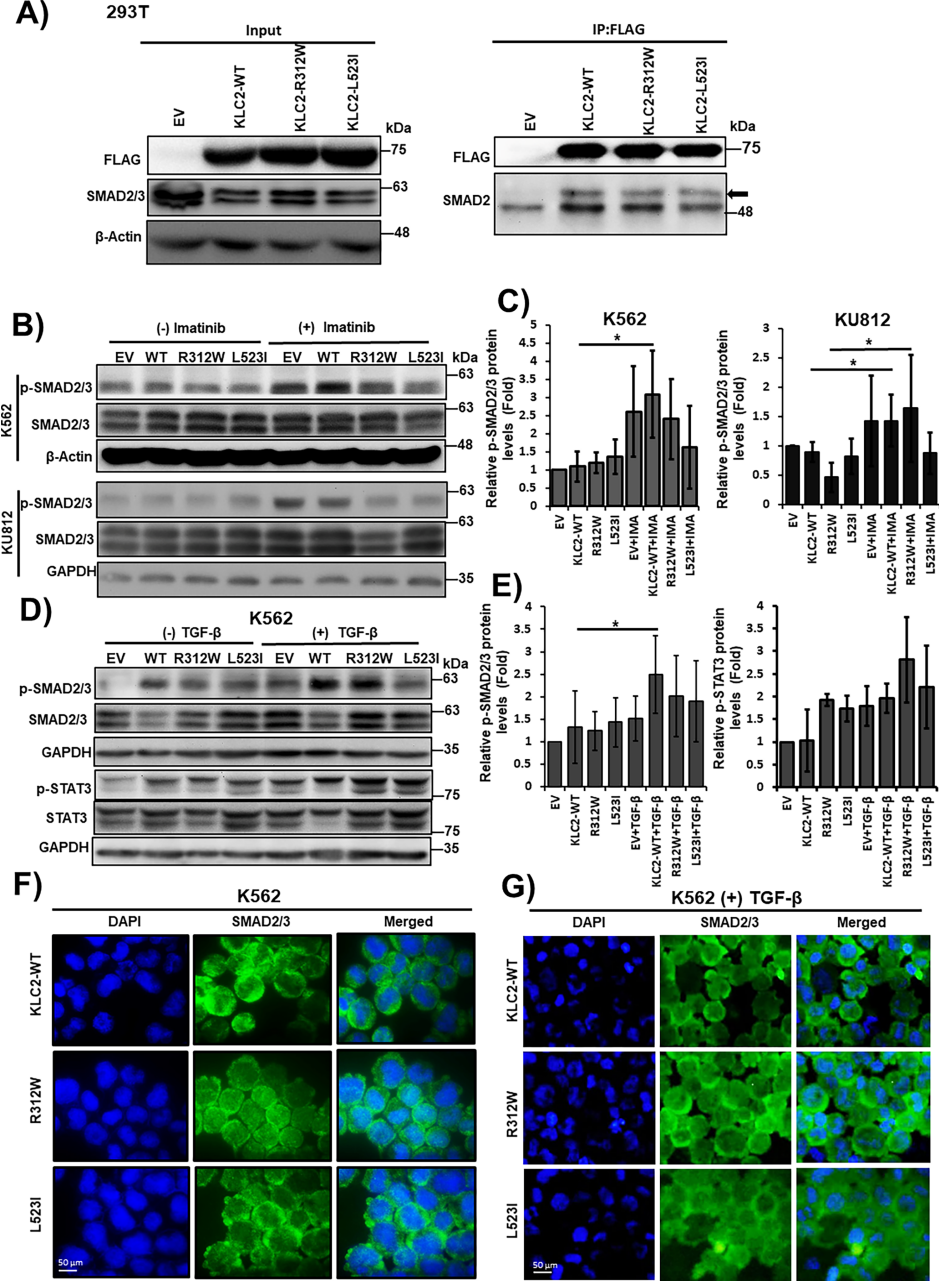
KLC2 mutants deregulate SMAD2/3 signaling in CML cells. (**A**) Transiently expressed FLAG-tagged KLC2-WT, KLC2-R312W, KLC2-L523I, and EV control in HEK293T cells, immunoprecipitation was performed using anti-FLAG antibody, and immunoblot was performed using a specific antibody. (**B**,**C**) KLC2-WT and MT-transduced stable K562 and KU812 cells were treated with 1 μm imatinib or without imatinib for 20 h and the indicated proteins were detected with specific antibodies by immunoblot analyses. (**D**,**E**) Similar K562 cells were starved overnight, then 10 ng/mL TGF-β was added for 1 h, indicated proteins were detected by immunoblotting. Non-treated EV cells were used as a control for the calculation of fold change. Either β-Actin or GAPDH is used for normalization. Error bars represent the mean ± SD (n ≥ 3), *p < 0.05. IMA indicated imatinib. (**F**,**G**) Subcellular localization of the SMAD2/3 proteins was visualized by immunofluorescence microscopy analysis in the transformed K562 cells without (**F**) and with (**G**) TGF-β treatment for 1 h. Scale bar = 50 μm

To determine whether KLC2-MT interacts with SMAD2, we performed a co-IP assay in HEK293T cells expressing *KLC2-*WT or *KLC2*-R312W/L523I MT and found that both MT interacted with SMAD2 (Fig. 4A). In addition, we observed activation of SMAD2/3 signaling in imatinib-treated K562 and KU812 CML cells; however, this activation was lower in L523I-expressing cells than in KLC2-WT cells (Fig. 4B,C). Furthermore, the KLC2 MT deregulated the transforming growth factor-β (TGF-β)–induced SMAD2/3 phosphorylation and STAT3 activation (Fig. 4D,E) in K562 cells. We performed immunofluorescence on transformed K562 cells with or without TGF-β treatment and immunoblotting without TGF-β treatment to assess the nucleocytoplasmic localization of SMAD2/3. However, we could not find a difference in the nucleocytoplasmic localization of SMAD2/3 between KLC2-WT and MT cells (Fig. 4F,G, Supplementary Fig. S13).

### 3.7 The Cooperation of KLC2 Mutants and BCR::ABL1 in Mouse BM Cells Increased Colony-Forming and Self-Renewal Activities

**Figure 5:**
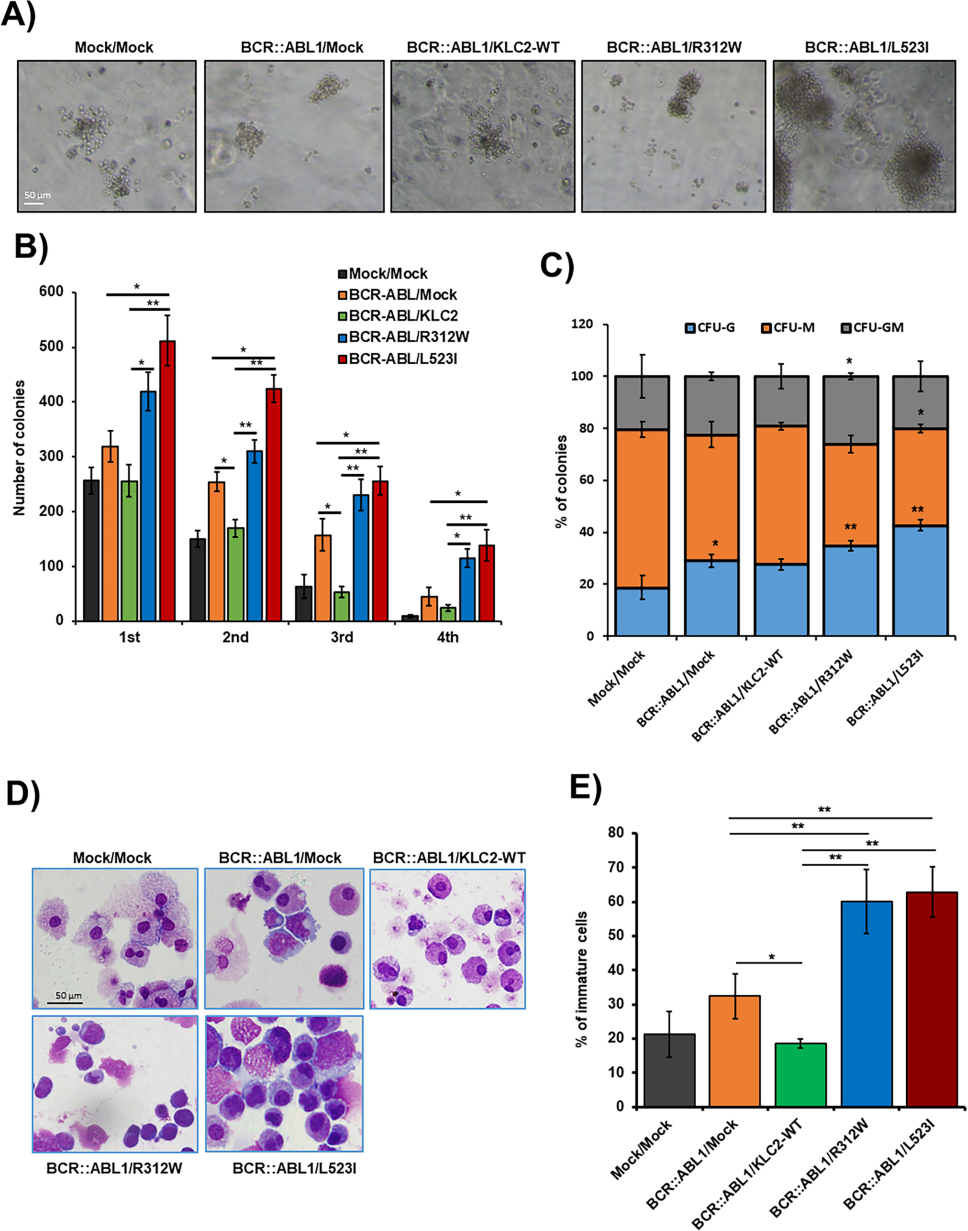
The cooperation of KLC2-R312W/L523I mutants and BCR::ABL1 in mouse BM cells increased colony-forming and self-renewal activities. (**A**,**B**) Colony-forming potentials of the cooperation of KLC2-R312W/L523I mutants and BCR::ABL1 in mouse BMCs (**A**). Scale bar = 50 μm. Columns (**B**) represent mean (sum of technical duplicates, mean of two independent experiments) ± SD, *p < 0.05, **p < 0.01. (**C**) The proportion of CFU-colonies of the 1st-round serial replating was shown. Columns represent the average number of types of colonies from duplicate cultures. *p < 0.05, **p < 0.01, compared with mock/mock. (**D**) Cytospin smeared preparations of cultured cells in the first plating colonies, as shown by Liu’s reagents staining, were shown. Scale bar = 50 μm. (**E**) Analysis of the number of immature cells in the first plating colonies. Error bars represent the mean ± SD (≥3) of different microscopic fields. *p < 0.05, and **p < 0.01

We transduced *BCR::ABL1* and *BCR::ABL1/KLC2-*WT/*KLC2-*R312W/*KLC2*-L523I, including EV constructs, in mouse BMCs using lentivirus-mediated gene transfer methods. We evaluated their colony-forming ability (Fig. 5A) and self-renewal activity (Fig. 5B) and determined the types of colonies, including colony-forming unit (CFU)–granulocyte (G), CFU–macrophage (M), and CFU–granulocyte–macrophage (GM) (Fig. 5C), in the MethoCult M3434 medium. We found that *BCR::ABL1/KLC2-*R312W/L523I–transduced BMCs had considerably better colony-forming ability with immature morphology (Fig. 5D,E) compared with either *BCR::ABL1* alone, *BCR::ABL/KLC2*-WT, or EV BMCs.

## 4 Discussion

Despite the high efficacy of TKIs in the treatment of *BCR::ABL1*-positive CML patients, a small portion of these patients demonstrate resistance and ultimately progress to the BP. Although a mutation in the BCR::ABL1 kinase domain is recognized as one of the major factors of TKI resistance and risk for blastic transformation, the processes behind CP-to-BP transformation are not completely understood. Recent studies [28–30] have reported the acquisition of additional genetic alterations during blastic transformation; among them, *KLC2* mutations have not been reported in the context of hematological neoplasms, which we sought to address for the biological role of KLC2-WT, KLC2-R312W, and KLC2-L523I mutations in CML-to-CML-BP transformation. The clinical relevance of *KLC2* mutation in relation to KLC2 expression in patient samples cannot be determined from the existing clinical dataset, as it is a novel finding with a very low mutation rate. Notably, *KLC2-*R312W and L523 mutations, but not N584 mutations, were registered in the COSMIC database. Among them, the L523 and N584 mutations in CML were recurrently found in C-terminal–proximal regions, indicating the functional importance of the C-terminal region of KLC2. The N-terminal heptad repeat region of KLC2 interacts with kinesin heavy chain, and the tetratricopeptide repeat domain interacts with cargo (Fig. S1) [18]. Thus, we decided to model KLC2-R312W and L523I MT located in the middle and end parts of KLC2 proteins, respectively, in the functional assay.

We expressed KLC2-WT, R312W, and L523I MT in CML cells *in vitro* and *in vivo* to examine the impact of KLC2 mutations in CML-to-CML-BP transformation. Consistent with the enhancement of cell growth and clonogenic potential, the self-renewal activity of primary murine BMCs was also increased in *KLC2*-R312W/L523I cells. Our findings from *in vitro* CML cells and a mouse xenograft model indicate that KLC2-MT may confer a proliferative advantage and impair apoptosis. *KLC2* knockdown from CML cells enhanced cell proliferation, decreased imatinib sensitivity, and reduced cleaved PARP-1, suggesting that KLC2 was required for the imatinib-induced apoptosis in CML cells. Consistent with a previous study [31], we observed the significant reduction in cell growth with the induction of apoptosis in *KLC2-*WT/MT–transduced K562 and KU812 cells in the presence of TKI. However, drug-induced apoptosis and cell growth inhibition were both reduced in *KLC2*-MT cells compared with *KLC2-*WT cells, suggesting that MT cells were more resistant to TKIs. Notably, the *KLC2*-L523I exhibited greater clonogenic and cell survival advantages in CML cells than R312W, indicating that the biological activities of the two MT were different. In fact, most of the biological activities of R312W mutation were different from those of L523I mutation and exerted a modest biological effect compared with control CML cells, indicating that mutation type and position potentially affect biological activities. We observed that imatinib had an insignificant effect on the xenograft model. For the xenograft study, we administered the drug when the tumors were palpable, in accordance with the report by Sanchez-Sanchez et al. [23]. We monitored the mice for 10 days post-tumor inoculation before administering imatinib. The delayed treatment initiation may have contributed to the limited efficacy observed in the xenograft experiment. In addition, the small cohort size constrained the statistical power of the xenograft study.

BCR::ABL1 enhances cell survival in CML by activating several signaling pathways, including mitogen-activated protein kinase (MAPK), AKT, and STAT5, which were downregulated with the TKI treatment [32–34]. Consistent with previous reports, MAPK, AKT, and STAT5 were downregulated in both *KLC2*-WT and MT CML cells with the imatinib or dasatinib treatment. Notably, imatinib or dasatinib increased Tyr705 phosphorylation of STAT3 (pSTAT3) in *KLC2-*WT as well as *KLC2*–R312W/L523I–expressing K562 and KU812 cells, and STAT3 activation in MT cells was higher than that in *KLC2-*WT cells. We measured the levels of phosphorylated STAT3 in KLC2-WT and MT CML cells after a 20 h drug treatment. However, we did not conduct a sequential study on the drugs to evaluate the p-STAT3 levels after the TKI treatment. This highlights a limitation of our study. The pSTAT3 levels did not decrease with the TKI treatment, suggesting that STAT3 activation does not depend on BCR::ABL1 activity, and additional genetic alterations may have been involved in CML-BP. Prior studies reported that aberrant protein transport and nucleocytoplasmic distribution had a substantial impact on cancer development and treatment response [35,36]. Nucleocytoplasmic shuttling is a classical nuclear transport process wherein a nuclear localization signal (NLS) is imported by β-karyopherin importin β directly or via the adaptor protein importin α, which is directly associated with classical NLS [37]. Phosphorylation of nucleocytoplasmic trafficking cargos enhances nuclear import by increasing their affinity for a specific import factor [38]. As KLC2 is a cargo protein that transports proteins involved in cell signaling [15,18], we hypothesized that KLC2-MT would deregulate CML cell growth and apoptosis by modulating STAT3 activation and subcellular localization. We observed nucleocytoplasmic shuttling of pSTAT3 in the presence of imatinib in CML cells and found that nuclear accumulation of activated STAT3 (pSTAT3) was greater in *KL*C2-MT cells than in *KLC2*-WT cells. Patel et al. emphasized that STAT3 promoted drug persistence through metabolic alterations in CML [39]. In addition, Eiring et al. reported that combined inhibition of STAT3 and BCR::ABL1 induced cell death in TKI-resistant CML [40]. The transcription factor, STAT3 plays a pivotal role in several hematological malignancies by regulating genes that control cell survival, proliferation, and metabolism [39,41,42]. Interestingly, a recent study reported a correlation between nucleocytoplasmic pSTAT3 levels and overall survival in patients with clear cell renal cell carcinoma [43]. Moreover, we found that pSTAT3 was more stable in *KLC2-*L523I MT cells than in *KLC2*-WT cells, indicating that the activation and nuclear accumulation of STAT3 were associated with the resistance of MT cells to imatinib. In addition, we demonstrated that *STAT3* knockdown decreased cell proliferation and increased imatinib sensitivity in *KLC2*-R312W and *KLC2*-L523I MT cells, similar to WT cells, highlighting the pivotal role of STAT3 in mediating drug resistance in CML cells. We also observed that silencing of *KLC2* from CML cells reduced the potency of imatinib with STAT3 activation. Our results indicated the important role of KLC2 in STAT3 regulation in CML cells, which may be deregulated by *KLC2* mutation.

**Figure 6:**
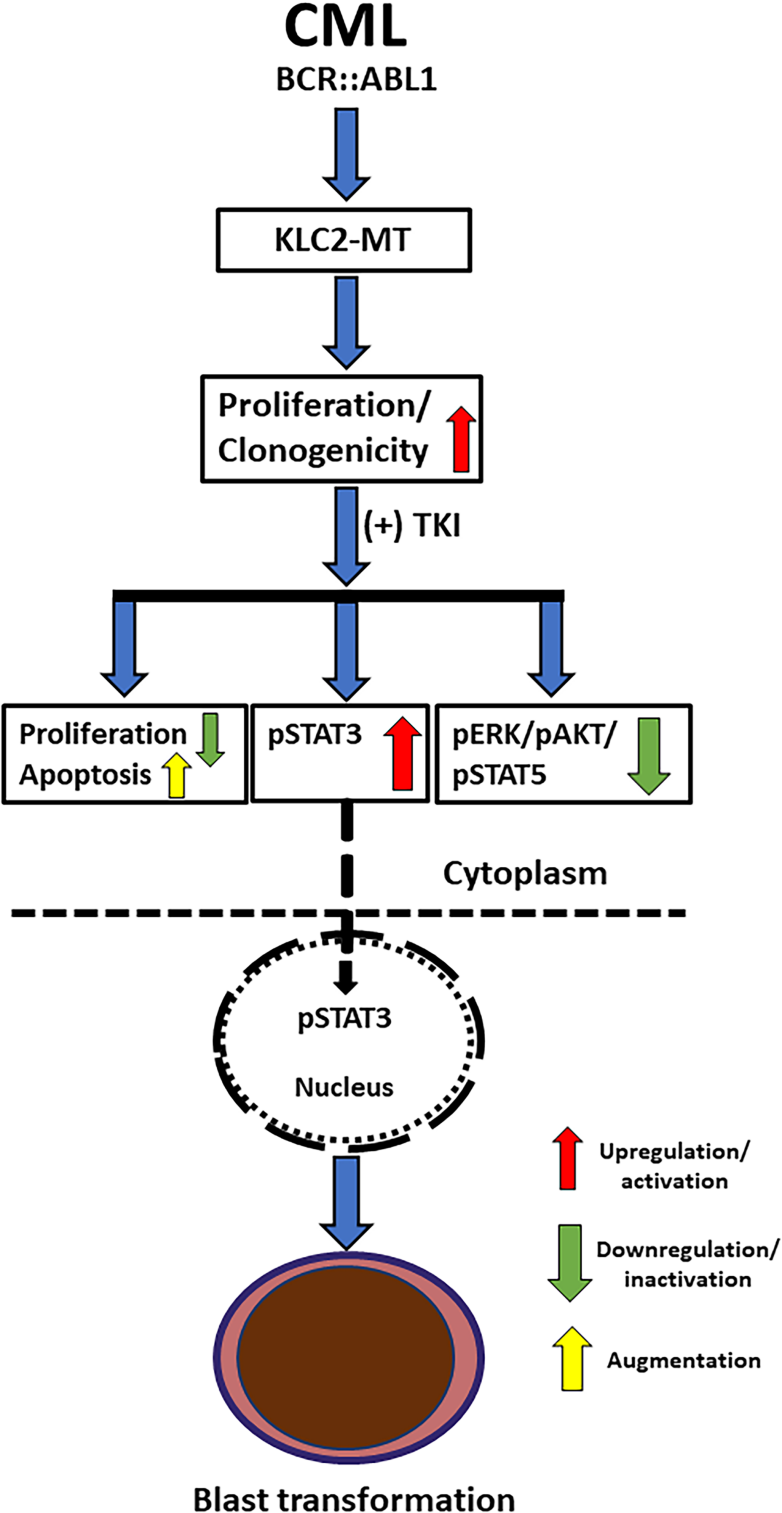
Schematic presentation of the crucial role of STAT3 in the blastic transformation of KLC2-mutated CML. Expression of KLC2-mutant enhanced cell proliferation, impaired TKI-induced apoptosis; together with the activated STAT3 (pSTAT3) and increased nuclear accumulation of pSTAT3 in the presence of TKI might promote the blast transformation of CML and drug resistance

The malignant phenotype and drug resistance of solid tumors are mediated by numerous kinesin proteins, including KLC2 [44]. Batut et al. reported that the C-terminal region of KLC2 is essential for binding to SMAD2, which is necessary for TGF-β signal transduction [18]. In the present study, KLC2*-*WT and R312W/L523I MT bound with SMAD2 and SMAD2/3 signaling were activated upon treatment with either imatinib or TGF-β in the transformed CML cells. However, SMAD2/3 signaling was less activated in L523I cells compared with *KLC2*-WT or R312W cells, despite modest STAT3 activation upon treatment with TGF-β. TGF-β inhibited the growth of hematopoietic stem and progenitor cells [45,46]. The TGF-β1/SMAD signaling pathway is crucial for cell growth inhibition, differentiation, and apoptosis in CML [47]. KLC2 was involved in the nuclear transport of SMAD2 [18], and TGF-β–mediated nucleocytoplasmic shuttling of SMAD2/3 regulated target gene transcription [36,48]. Furthermore, SMAD3 physically interacted with STAT3 [49,50], and STAT3 signaling modulated TGF-β–mediated transcriptional and biological responses, including cell growth, cell cycle arrest, and cell apoptosis [50]. We observed that nuclear transportation of SMAD2/3 was comparable in CML cells transduced with either *KLC2-*WT or KLC2-MT. Therefore, aberrant activation and nuclear accumulation of pSTAT3 are potentially critical for imatinib resistance in *KLC2-*MT CML cells (Fig. 6). The mislocalization of STAT3 and the role of SMAD2 in the nucleocytoplasmic shuttling of STAT3 via the nuclear transport machinery in KLC2-MT cells remain unclear. Consequently, the mechanisms by which KLC2 mutations enhance STAT3 activation in CML cells or whether this activation relies on the KLC2/SMAD2/3 axis remain unclear, and we acknowledge these limitations.

## 5 Conclusion

To the best of our knowledge, this is the first to identify the biological importance of the *KLC2* mutation in CML cells, which could be a risk biomarker of CML with blastic transformation. We demonstrated that TKI-mediated STAT3 phosphorylation and nuclear accumulation via KLC2 abnormalities may play a role in CML progression. Our findings may provide a better understanding of the disease progression and facilitate the development of effective treatments for a subset of CML patients carrying these mutations.

## Supplementary Materials





























## Data Availability

All data generated or analyzed during this study are included in this article and its supplementary information files. Raw and processed data are available upon reasonable request.

## Supplementary Materials

The supplementary material is available online at https://www.techscience.com/doi/10.32604/or.2025.070259/s1.
