# Supplementary figures and images for: Biological Features of KLC2 Mutations in Chronic Myeloid Leukemia and Their Contribution to Inducing Drug Resistance
Source: Oncol Res. 2025 Dec 30;34(1):10. doi: 10.32604/or.2025.070259 (PMC12774541; doi:10.32604/or.2025.070259)

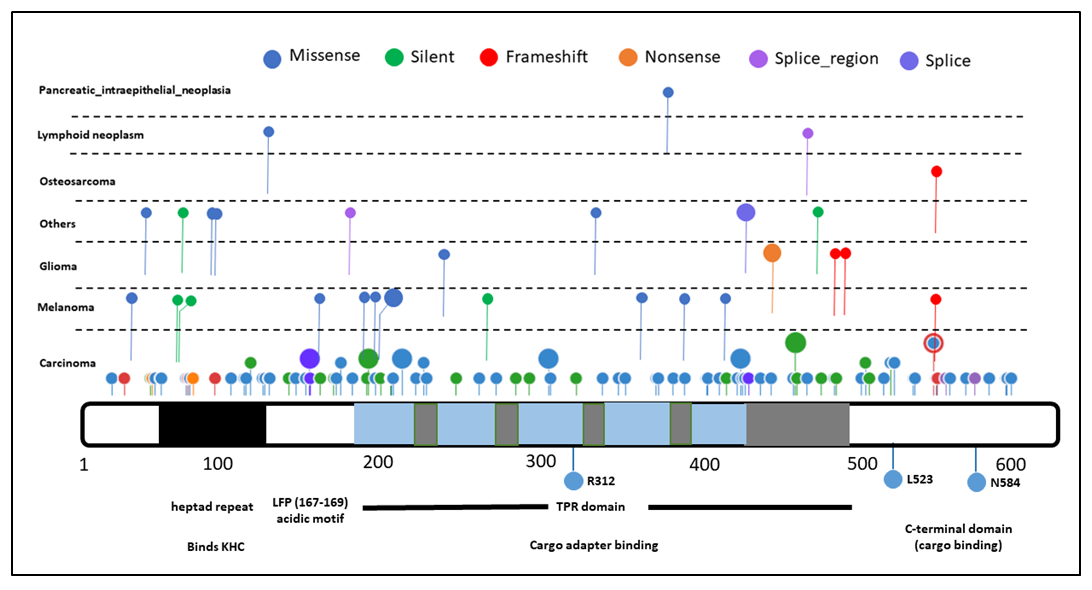

Supplement: Supplementary file 1 [file OncolRes-34-70259-s001.tif]

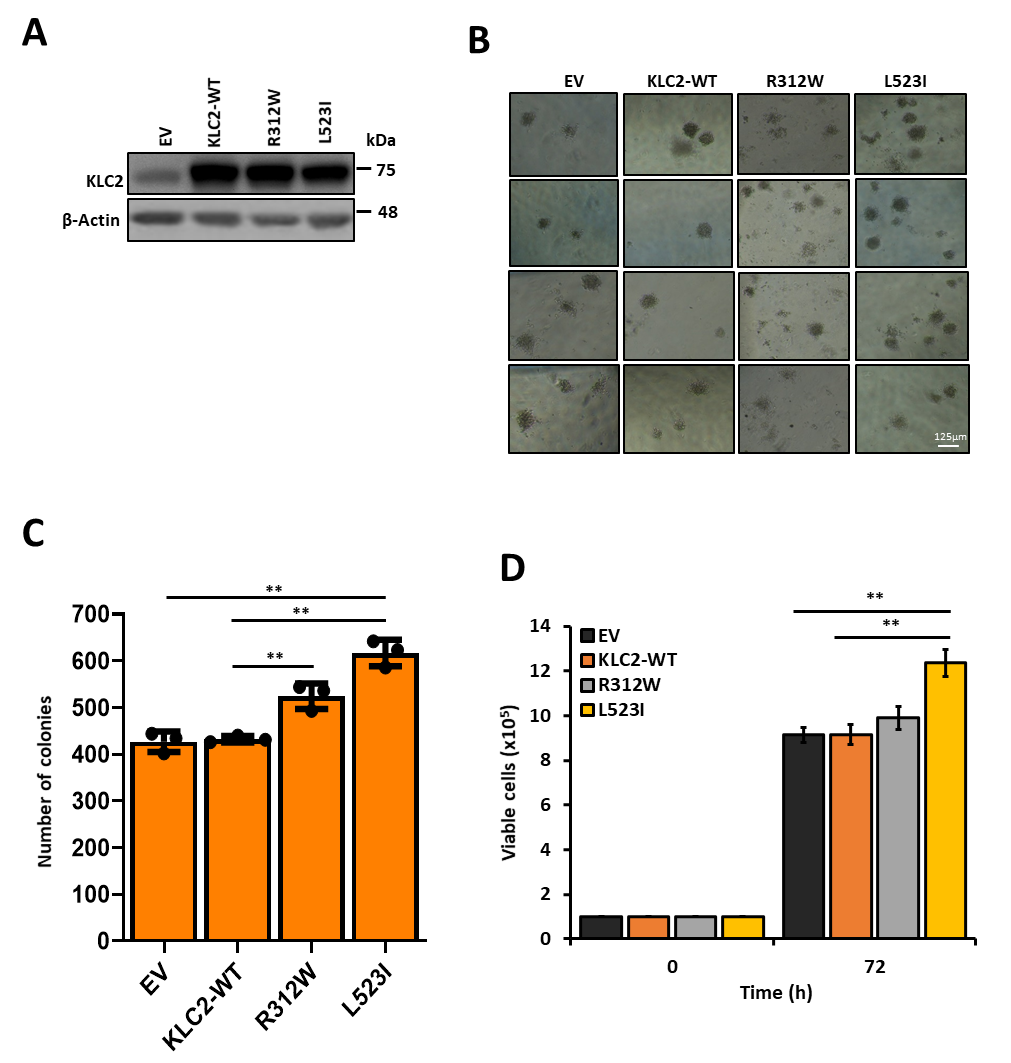

Supplement: Supplementary file 2 [file OncolRes-34-70259-s002.tif]

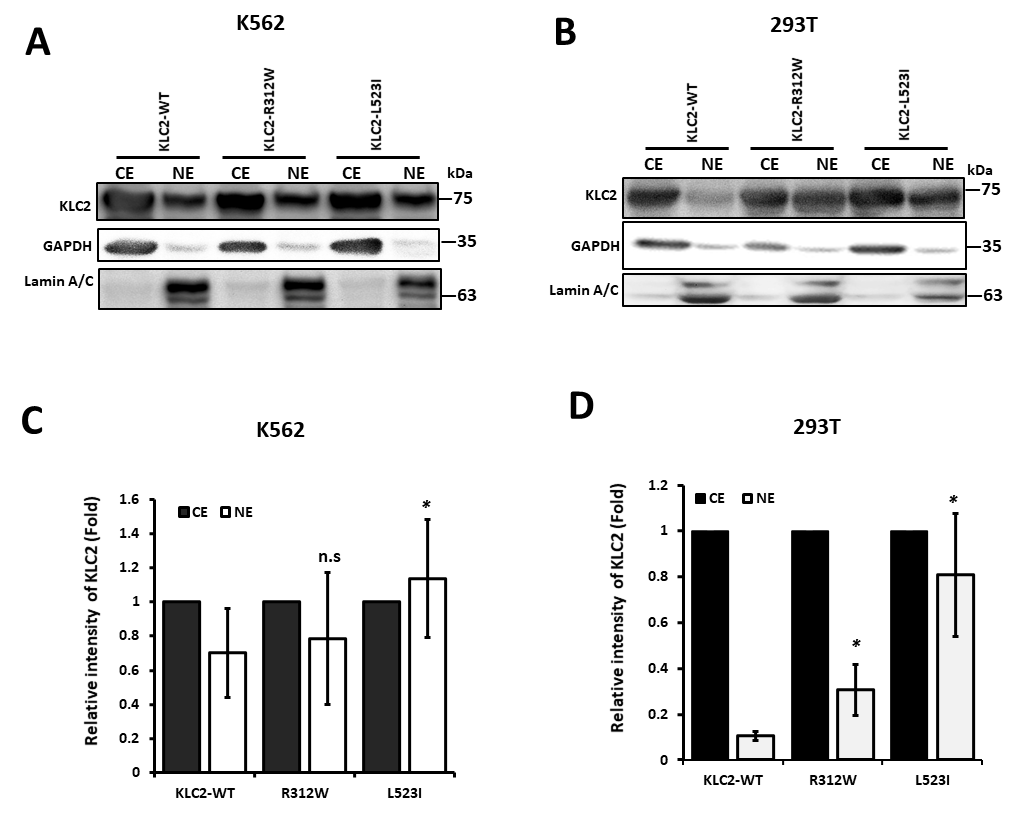

Supplement: Supplementary file 3 [file OncolRes-34-70259-s003.tif]

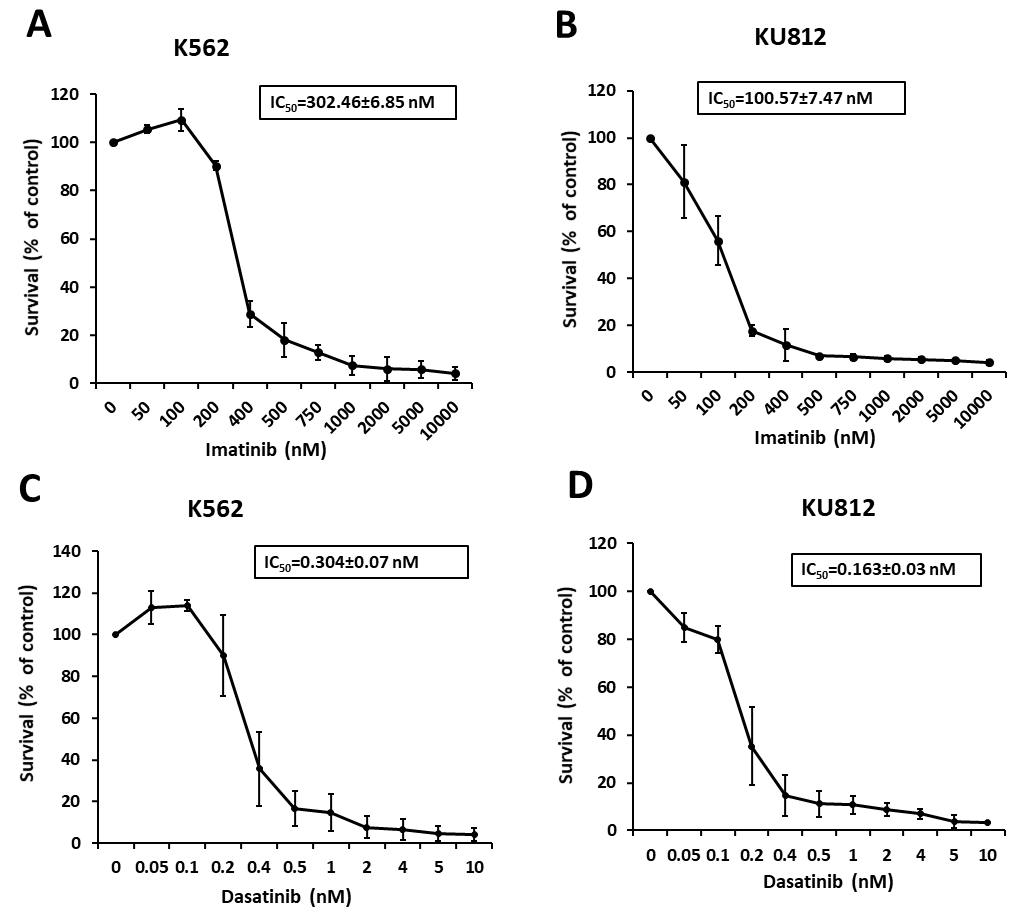

Supplement: Supplementary file 4 [file OncolRes-34-70259-s004.tif]

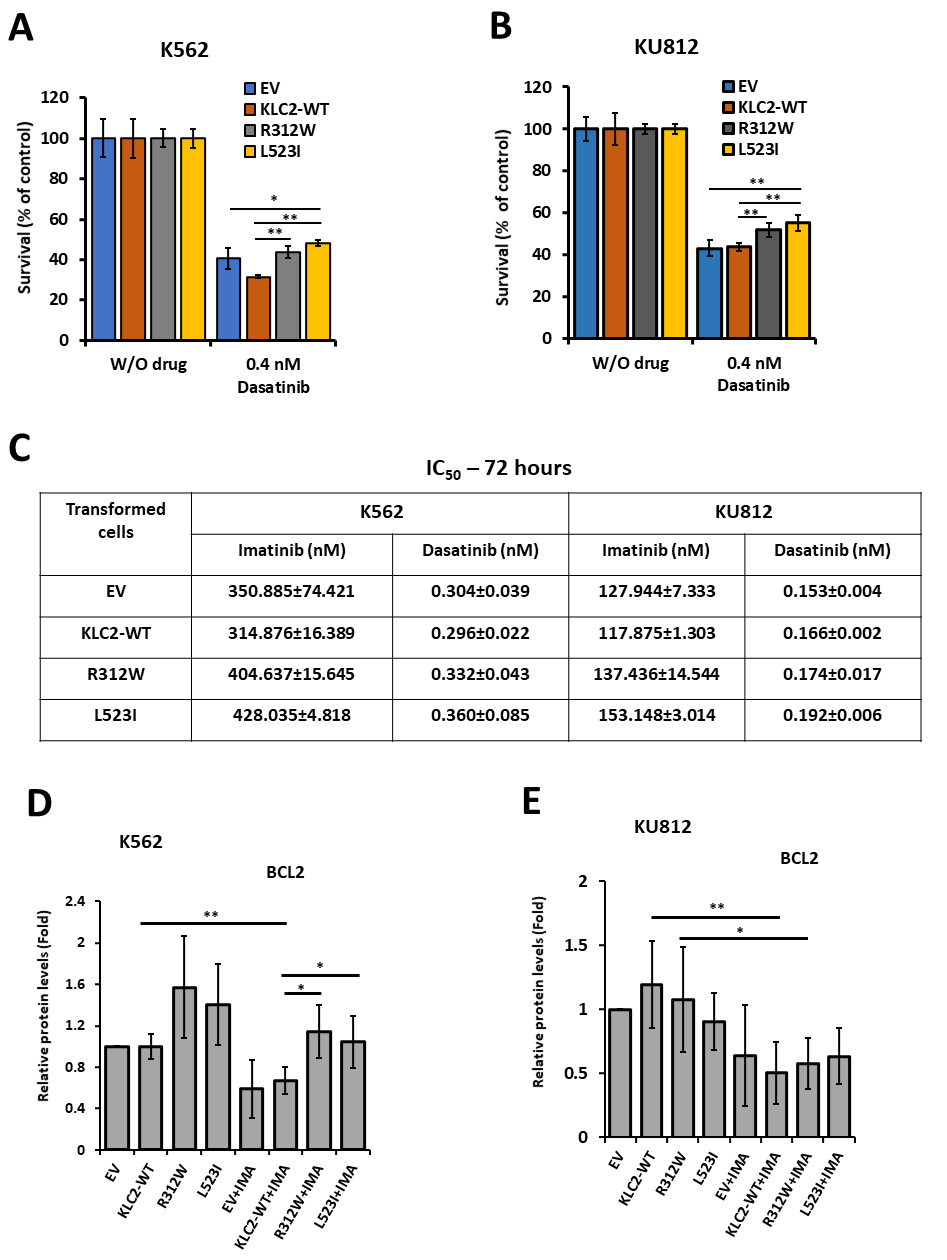

Supplement: Supplementary file 5 [file OncolRes-34-70259-s005.tif]

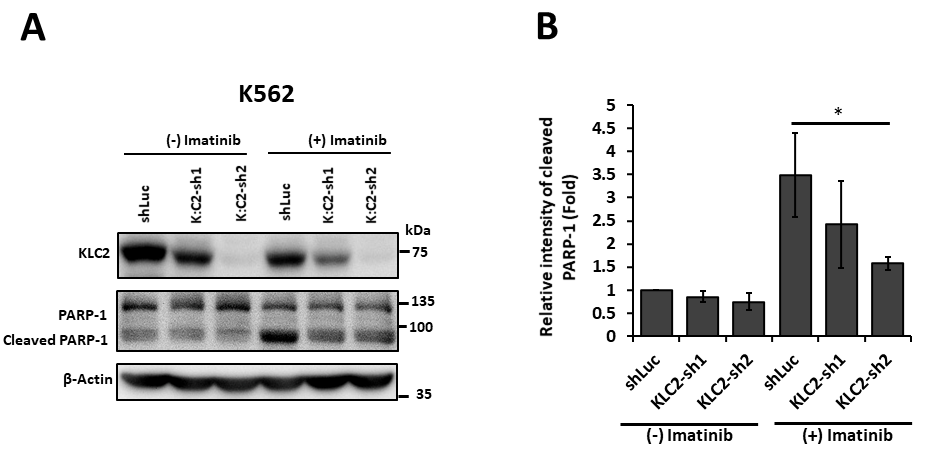

Supplement: Supplementary file 6 [file OncolRes-34-70259-s006.tif]

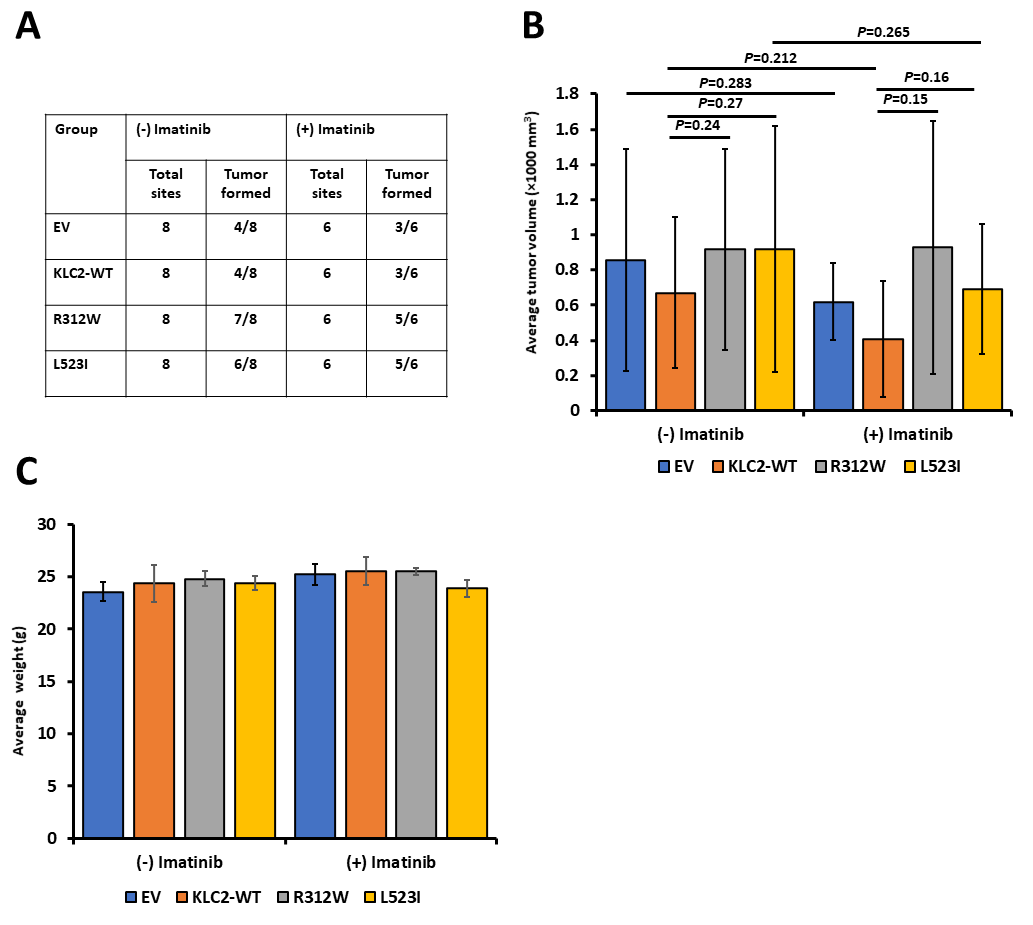

Supplement: Supplementary file 7 [file OncolRes-34-70259-s007.tif]

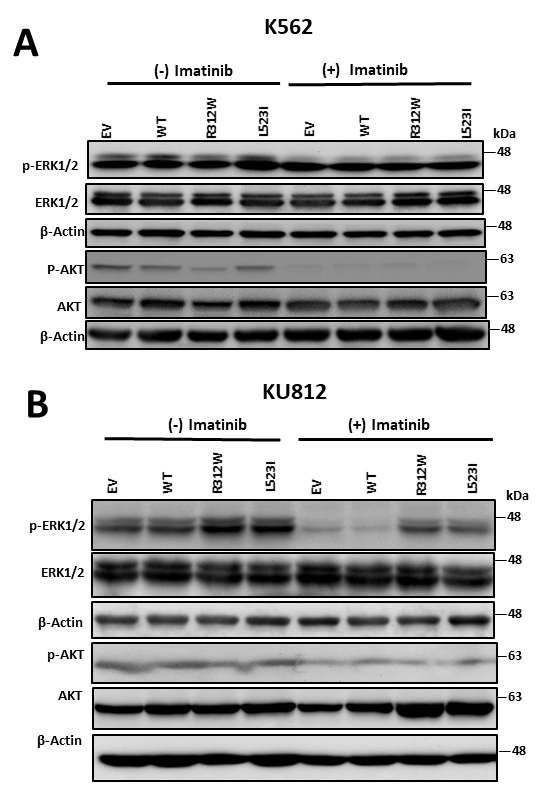

Supplement: Supplementary file 8 [file OncolRes-34-70259-s008.tif]

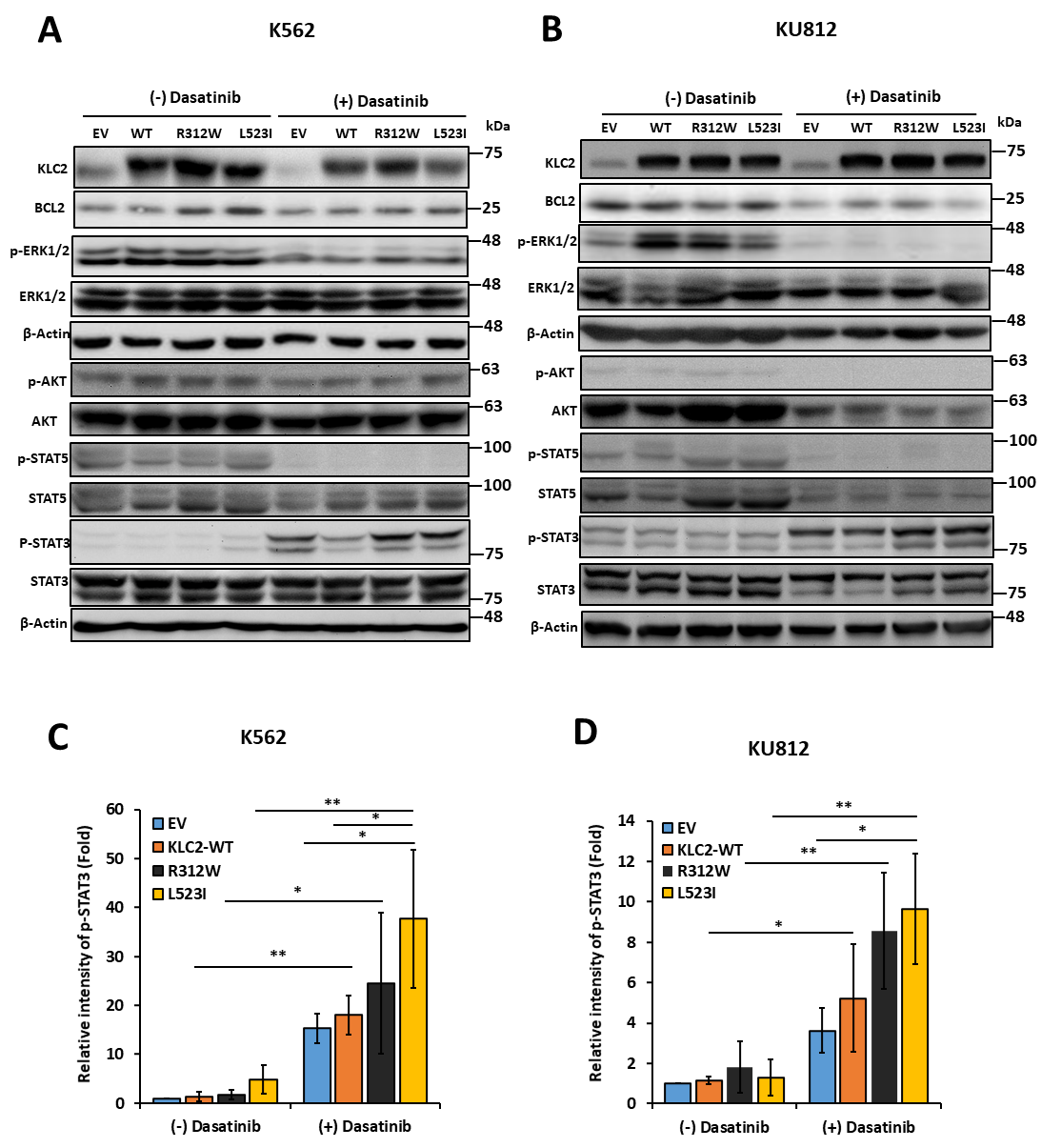

Supplement: Supplementary file 9 [file OncolRes-34-70259-s009.tif]

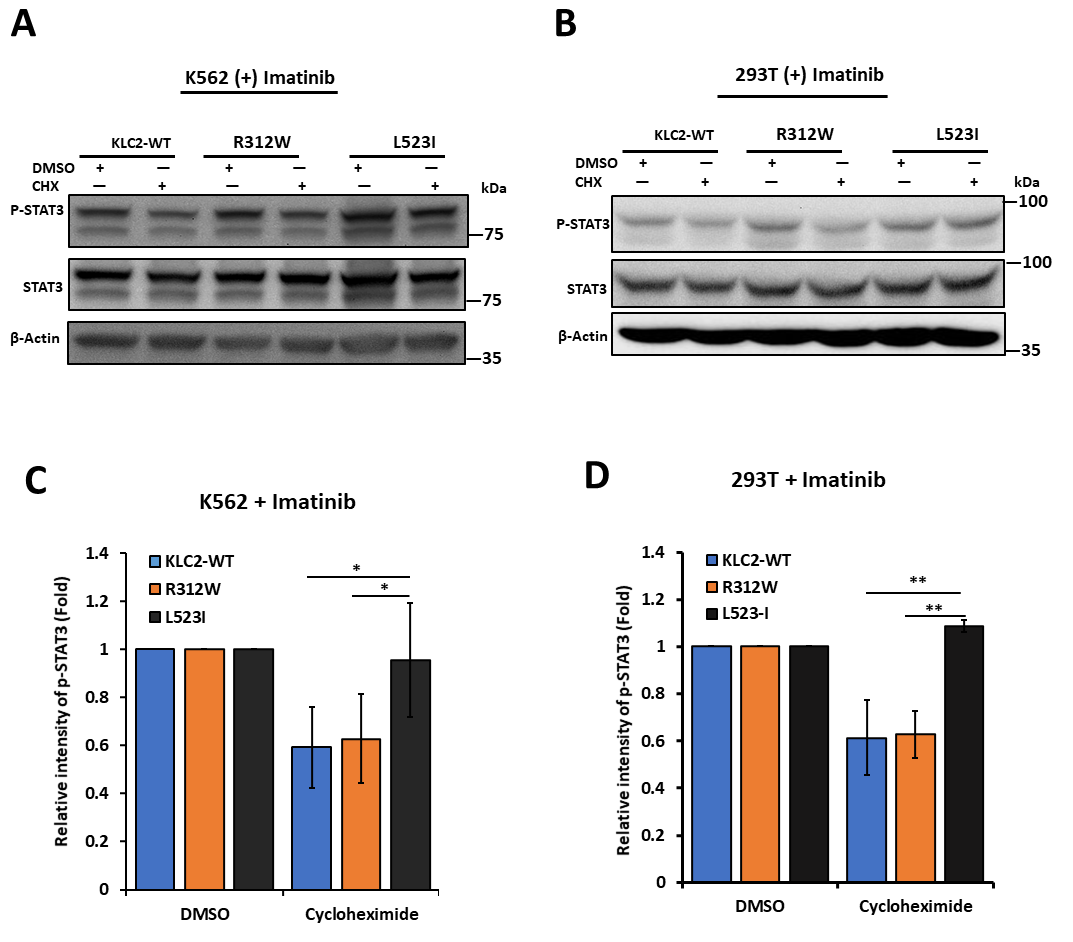

Supplement: Supplementary file 10 [file OncolRes-34-70259-s010.tif]

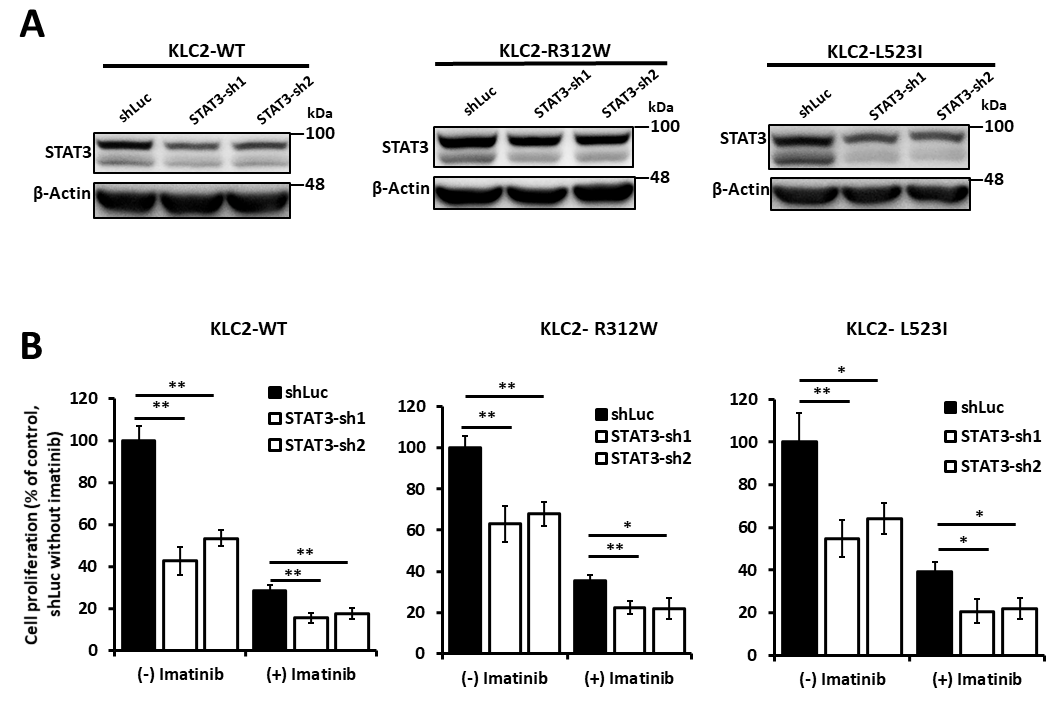

Supplement: Supplementary file 11 [file OncolRes-34-70259-s011.tif]

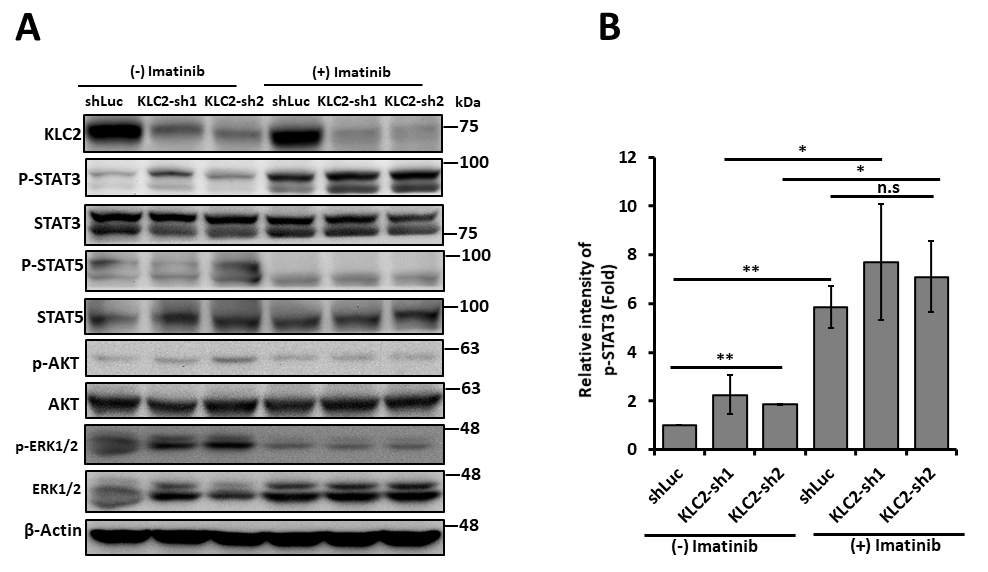

Supplement: Supplementary file 12 [file OncolRes-34-70259-s012.tif]

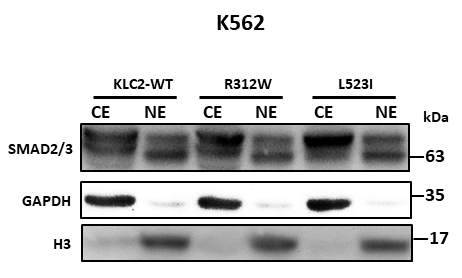

Supplement: Supplementary file 13 [file OncolRes-34-70259-s013.tif]
