# Supplementary material for: Biological Features of KLC2 Mutations in Chronic Myeloid Leukemia and Their Contribution to Inducing Drug Resistance
Source: Oncol Res. 2025 Dec 30;34(1):10. doi: 10.32604/or.2025.070259 (PMC12774541; doi:10.32604/or.2025.070259)
Supplement: Supplementary file 14 [file OncolRes-34-70259-s014.pdf]

SUPPLEMENTARY MATERIALS

Biological features of KLC2 mutations in chronic myeloid leukemia and their contribution to inducing drug resistance

Bera R, Ochi Y, Huang Y-J, Kuo M-C, Yoshida K, Ogawa S, and Shih L-Y

1. Supplementary Methods

Plasmid construction, lentiviral preparation, and infection

For this study, we selected the KLC2-R312W and L523I mutations, which are located in the middle- and C-terminal–proximal regions of the KLC2 proteins, respectively. The full-length cDNA of human KLC2-WT with FLAGtag was constructed into the EcoRI/BamHI multiple cloning sites of lentiviral vector pCDH1-MSCV-MCS-EF1-Puro according to the standard method and verified by sequencing. Point mutants R312W and L523I of the *KLC2* gene were generated from FLAG-KLC2-WT using site-directed mutagenesis (KAPA HiFi HotStart, Kapa Biosystems, Wilmington, MA, USA) and confirmed by full-length DNA sequencing. KLC2-WT and mutants tagged with a FLAG epitope at the N-terminus were subcloned into pIRES2-EGFP-vector. Similarly, BCR-ABL1 (p210) with FLAGtag was constructed into the EcoRI/NotI multiple cloning sites of lentiviral vector pCDH-MSCV-Puro and pCDH-CMV-CoEGFP plasmids, respectively. All sequences were confirmed by direct sequencing before expression in cells. The pLKO.1-puro plasmid-based shRNAs, including Luc (luciferase shRNA, TRCN231719), human KLC2-sh1 (TRCN0000155828), and KLC2-sh2 (TRCN0000155929); human STAT3-sh1 (TRCN0000329886) and STAT3-sh2 (TRCN0000020842), were obtained from the National RNAi Core laboratory, Taiwan. 293T cells transfection was carried out using Lipofectamine® 3000 reagent (Invitrogen; Thermo Fisher Scientific, Inc., Waltham, MA, USA) according to the manufacturer's protocol. K562, KU812, and U937 cultured cells were spun infected in the presence of 8 µg/mL polybrene (Sigma-Aldrich) and cultured for 60 h. Lentivirus-infected cells were selected with 2µg/mL puromycin (101-58-58-2, MD Bioscience Inc., MN, USA) to prepare stable cell lines.

2. Supplementary Tables

Supplementary Table S1: List of antibodies for immunoblots and immunofluorescence staining

Application	Name	Catalog number	Company	Dilution
Immunofluorescence	KLC2	17668-1-AP	Proteintech, Rosemont, IL , USA	1:100
	STAT3	10253-AP	Proteintech, Rosemont, IL , USA	1:100
	Phospho-STAT3 (Tyr705)	9145	Cell Signaling Technology, Danvers, MA, USA	1:100
	SMAD2/3 (D7G7)	8685	Cell Signaling Technology, Danvers, MA, USA	1:200
Immunoblots	Phospho-AKT (Ser473)	9271	Cell Signaling Technology, Danvers, MA, USA	1:1000
	Phospho-STAT5 (Tyr694)	9351	Cell Signaling Technology, Danvers, MA, USA	1:1000
	Phospho-ERK	4370	Cell Signaling Technology, Danvers, MA, USA	1:1000
	γ-H2A.X (Ser139)	2577	Cell Signaling Technology, Danvers, MA, USA	1:1000
	Phospho-STAT3 (Tyr705)	9145	Cell Signaling Technology, Danvers, MA, USA	1:1000
	SMAD2/3 (D7G7)	8685	Cell Signaling Technology, Danvers, MA, USA	1:1500
	KLC2	17668-1-AP	Proteintech, Rosemont, IL , USA	1:2000
	STAT3	10253-AP	Proteintech, Rosemont, IL , USA	1:1000
	AKT	10176-2-AP	Proteintech, Rosemont, IL , USA	1:1000
	STAT5A	13179-1-AP	Proteintech, Rosemont, IL , USA	1:1000
	Lamin-A/C	10298-1-AP	Proteintech, Rosemont, IL , USA	1:1000
	BCL2	12789-1-AP	Proteintech, Rosemont, IL , USA	1:1500
	Phospho-Smad2-S465/467 + Smad3-S423/425	AP0548	Abclonal, Woburn, MA, USA	1:1000
	FLAG	F3165	Sigma Aldrich, St. Louis, MO, USA	1:1000
	β-Actin	A5441	Sigma Aldrich, St. Louis, MO, USA	1:2000
	Histone 3	ab70550	Abcam, Cambridge, UK	1:1000
	PARP-1	GTX100573	GeneTex, Irvine, CA, USA	1:1000
	GAPDH	GTX100118	GeneTex, Irvine, CA, USA	1:2000
	ERK1	sc-94	Santa Cruz Biotechnology, Texas, USA	1:1000

	HRP-anti-mouse IgG	115-035-003	Jackson ImmunoResearch, West Grove, PA, USA	1:10000
	HRP-anti-rabbit IgG	111-035-003	Jackson ImmunoResearch, West Grove, PA, USA	1:10000
	FITC-conjugated anti-mouse IgG	115-095-00	Jackson ImmunoResearch, West Grove, PA, USA	1:200
	FITC-conjugated anti-rabbit IgG	111-095-003	Jackson ImmunoResearch, West Grove, PA, USA	1:200

Supplementary Table S2: The information regarding 3 mutations in *KLC2*

Sample ID	Chr	Start	End	Ref	Alt	Gene	Type_ mutation	AA change	VAF
TW_CML_M_006_BC	11	66033927	66033927	A	G	*KLC2*	nonsynonymous SNV	KLC2:NM_001134774:exon14:c.A1520G:p.N507S, KLC2:NM_001134775:exon15:c.A1751G:p.N584S, KLC2:NM_001134776:exon15:c.A1751G:p.N584S, KLC2:NM_022822:exon15:c.A1751G:p.N584S	0.487
TW_CML_M_065_BC	11	66033448	66033448	C	A	*KLC2*	nonsynonymous SNV	KLC2:NM_001134774:exon12:c.C1336A:p.L446I,KLC2:NM_001134775:exon13:c.C1567A:p.L523I,KLC2:NM_001134776:exon13:c.C1567A:p.L523I,KLC2:NM_022822:exon13:c.C1567A:p.L523I	0.36
TW-CML-M-079-BC	11	66031415	66031415	C	T	*KLC2*	nonsynonymous SNV	KLC2:NM_001134774:exon6:c.C703T:p.R235W,KLC2:NM_001134775:exon7:c.C934T:p.R312W,KLC2:NM_001134776:exon7:c.C934T:p.R312W,KLC2:NM_022822:exon7:c.C934T:p.R312W	0.024

3. Supplementary Figures

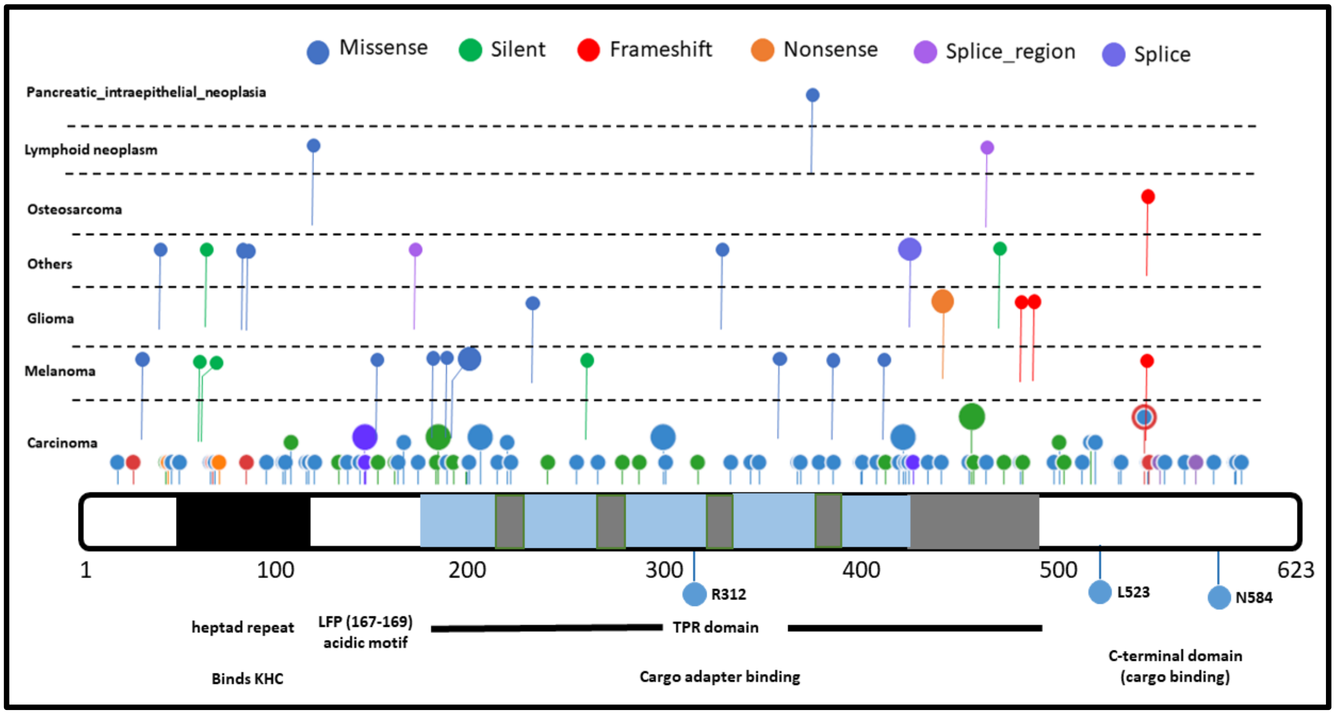

Figure S1

Figure S1. Distributions of the KLC2 mutations in CML and other human cancers from COSMIC. Schematic diagram of novel *KLC2* mutations in CML (downside) and other human cancers (upper side) drawn using COSMIC database. *KLC2* mutations in human cancers distributed in the protein domains are indicated in the same diagram. Schematics of the KLC2; tetratricopeptide repeat (TPR), kinesin heavy chain (KHC), leucine– phenylalanine–proline (LFP) motif.

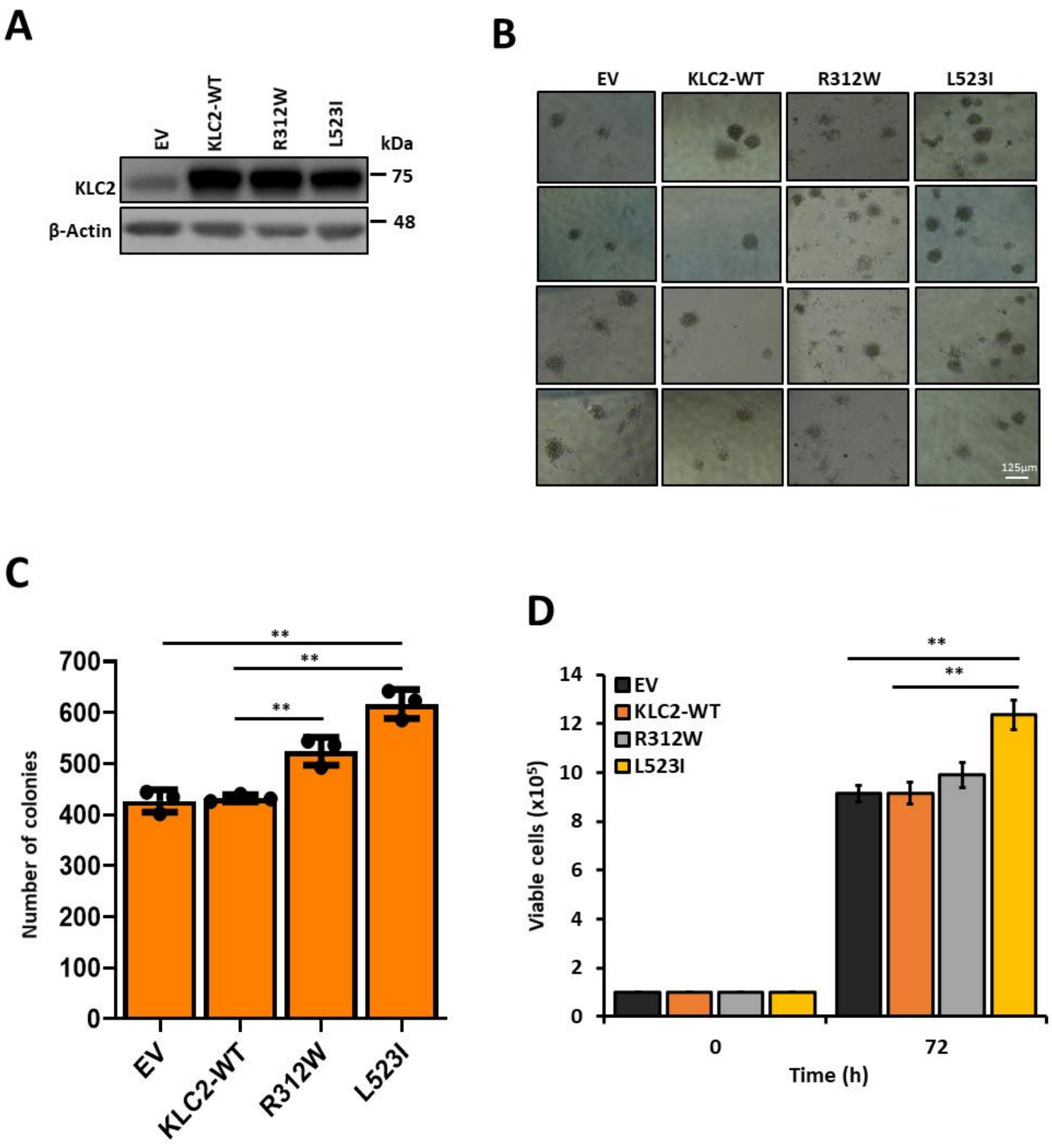

Figure S2

Figure S2. KLC2 mutants provide proliferation advantage and clonogenic potential in U937 cells (related to Figure 1). (A) KLC2-WT, R312W, and L523I expressions were checked in stably transformed U937 cells by Immunoblotting. β-Actin was used as a control for equal loading. (B–C) Colony-forming assay was performed after the stable expression of KLC2-WT and mutant cells. Scale bar = 125 µm. Columns represent mean (sum of technical triplicates, mean of three independent experiments) ± SD; ***P*<0.01. (D) U937 transformed cells were grown for 72 h and viable cells were counted by the trypan blue exclusion method. Error bars represent the mean ± SD of three independent experiments; ***P*<0.01.

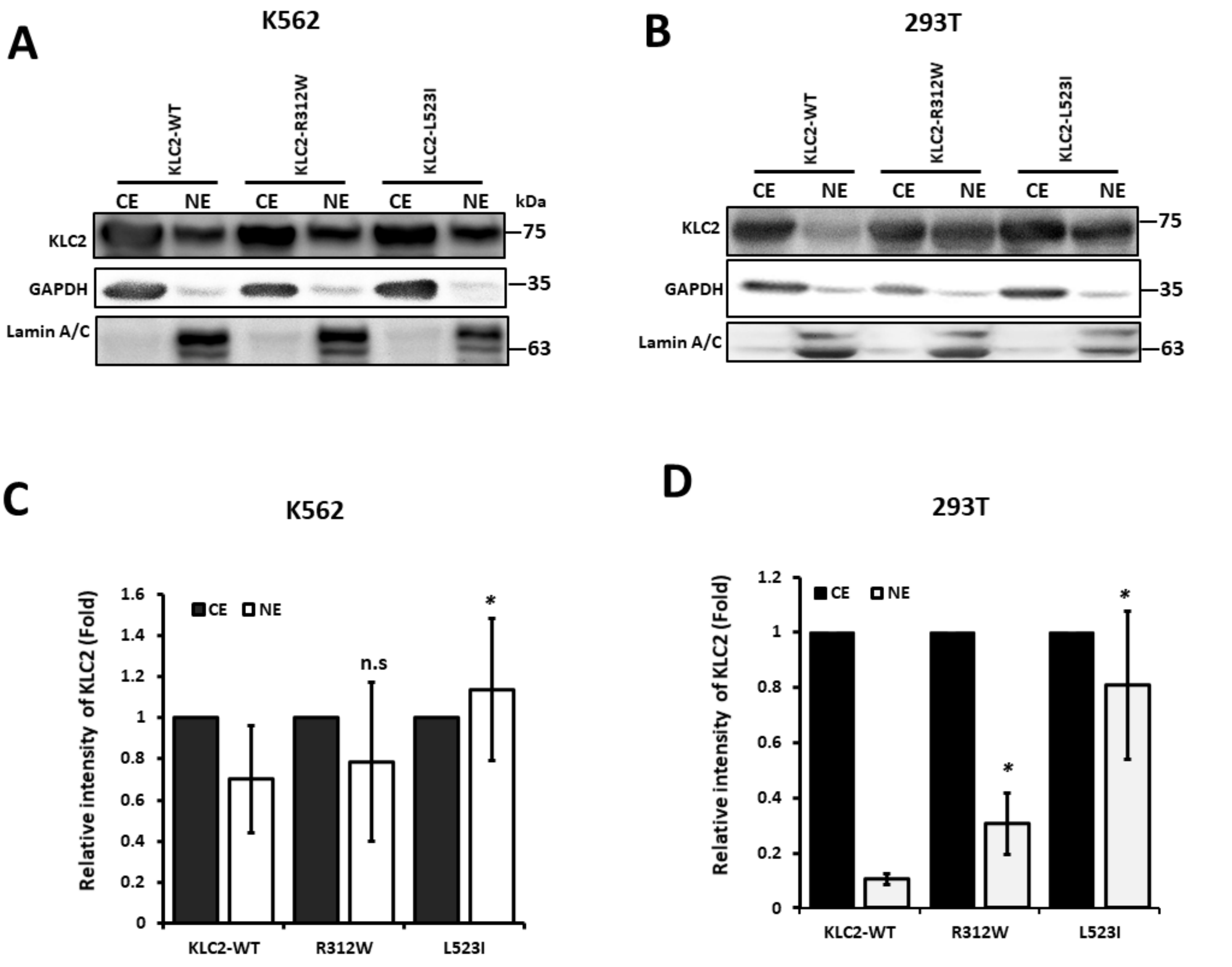

Figure S3

Figure S3. Subcellular localization of KLC2-WT/mutants in transformed cells (related to Figure 1). (A-B) KLC2-WT and mutants were stably expressed in K562 (A) and transiently expressed in 293T cells (B). The cytoplasmic and nuclear distribution of KLC2-WT/MT protein was stepwise separated from similar K562 (A) and 293T (B) cells, analyses by Immunoblot using antibodies as indicated. (C-D) Cytoplasmic and nuclear proteins are quantified by densitometric analyses and presented in fold change of NE compared to CE. CE indicates cytoplasmic protein extract, and NE, nuclear protein extract. Error bars represent the mean ± SD of four (C**)** and three (D) independent experiments; **P*< 0.05 relative to KLC2-WT. n.s, not significant.

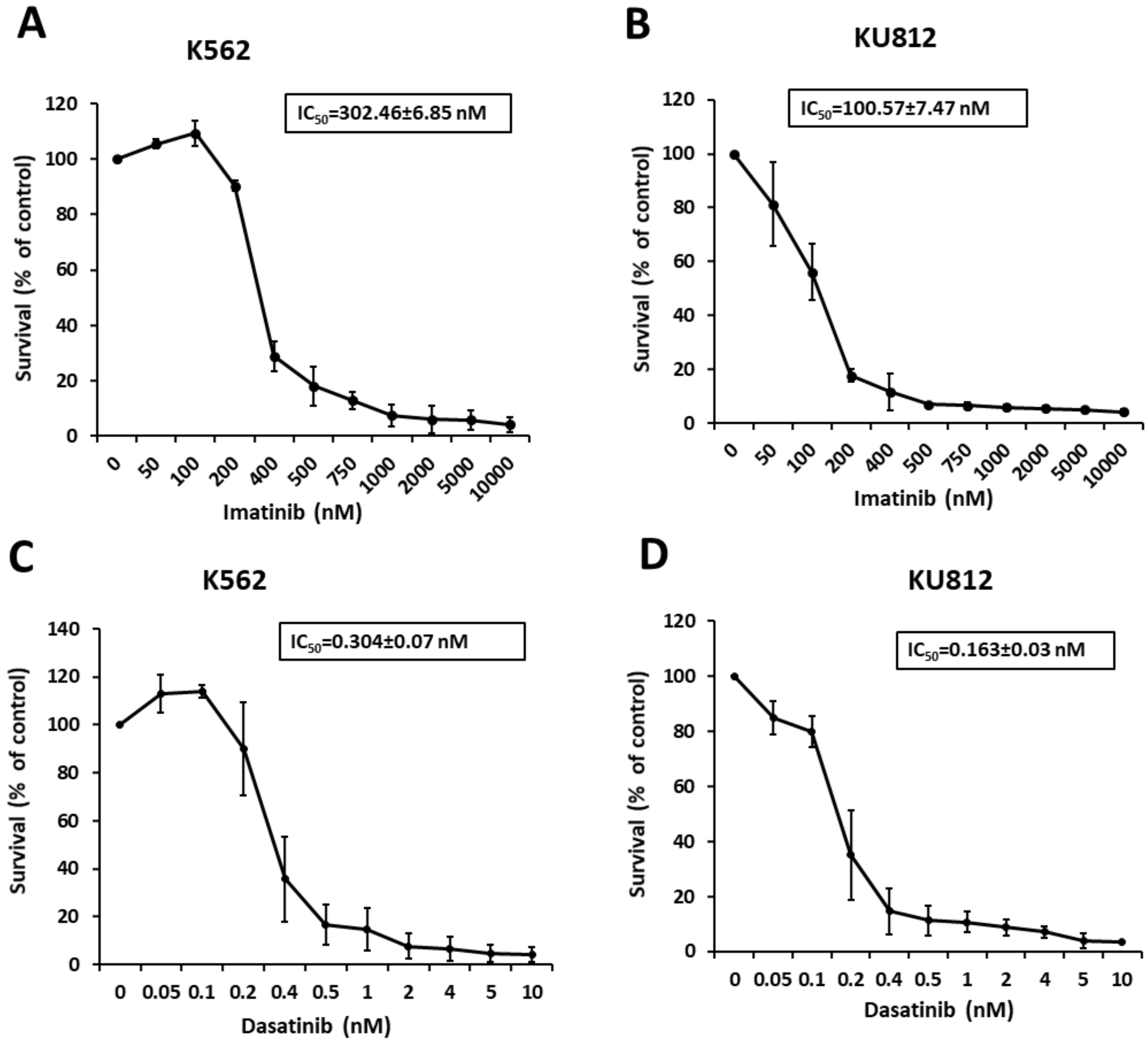

Figure S4

Figure S4. **Dose-dependent survival of K562 and KU812 cells in the presence of imatinib and dasatinib (related to Figure 2)**. K562 and KU812 cells were treated with different concentrations of imatinib (A–B) and dasatinib (C–D) in triplicate in a 96-well cell culture plate for 72 h, and viable cells were analyzed by CCK-8 reagents according to the manufacturer's protocol (Life Science Technologies). Cells without treatment with drugs were considered the control and IC_{50} values were calculated. Error bars represent the mean ± SD of two independent experiments. IC_{50} values are shown in the inset of each figure.

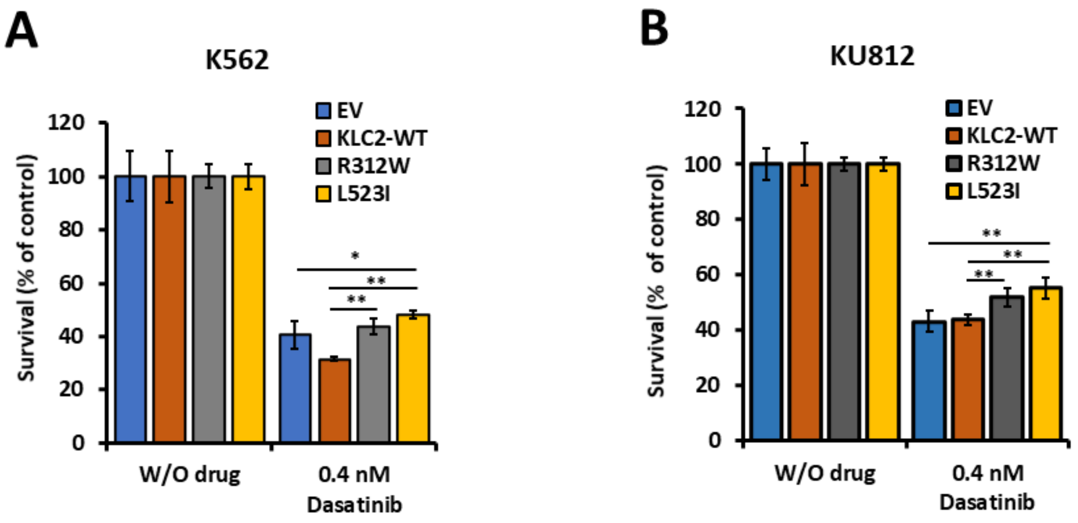

C

IC_{50} – 72 hours

Transformed cells	K562		KU812	
	Imatinib (nM)	Dasatinib (nM)	Imatinib (nM)	Dasatinib (nM)
EV	350.885±74.421	0.304±0.039	127.944±7.333	0.153±0.004
KLC2-WT	314.876±16.389	0.296±0.022	117.875±1.303	0.166±0.002
R312W	404.637±15.645	0.332±0.043	137.436±14.544	0.174±0.017
L523I	428.035±4.818	0.360±0.085	153.148±3.014	0.192±0.006

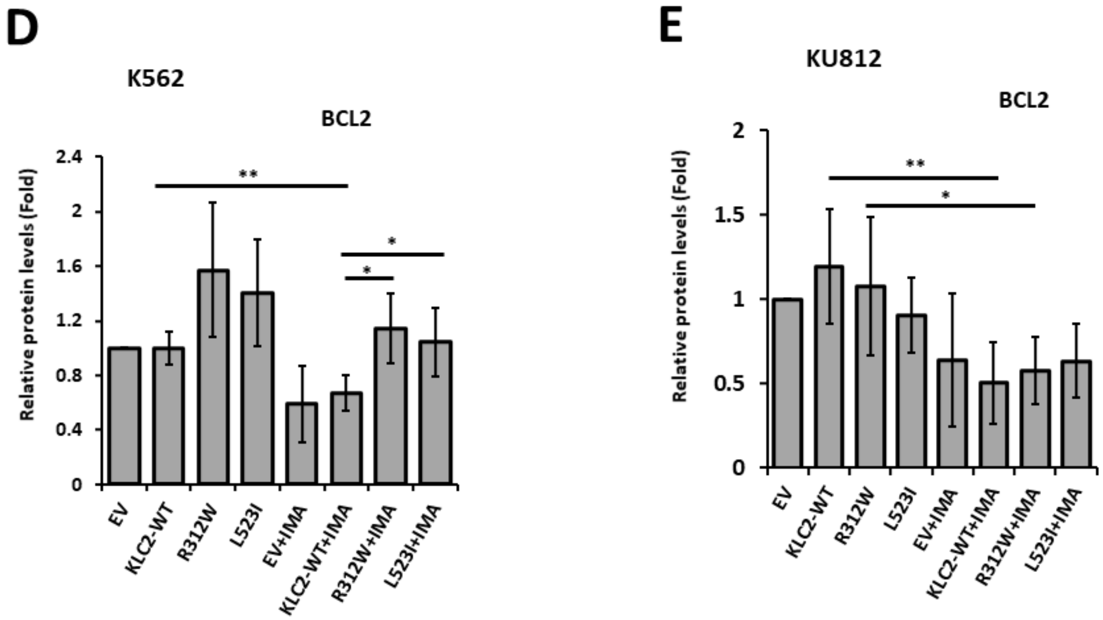

Figure S5

Figure S5. KLC2 mutants decreased the efficacy of CML cells to TKI and impaired imatinib-induced apoptosis *in vitro* (related to Figure 2). (A–B) KLC2-WT and mutant-expressing K562 (A) and KU812 (B) cells were treated with 0.4 nM dasatinib or without drugs for 72 h; the cell survival rate was evaluated using the trypan blue exclusion method. Error bars represent the mean ± SD of three independent experiments, **P*<0.05, ***P*<0.01. (C) Transformed K562 and KU812 cells were treated without drugs or with the different doses of imatinib and dasatinib for 72 h, and viable cells were analyses by CCK-8 reagents. The IC_{50} value is shown in the figure (C) and represents the mean ± SD of two independent experiments. (D–E) K562 cells were treated with Imatinib (400 nM) (C) and KU812 cells with 100 nM imatinib for 72 h or without imatinib and BCL2 protein level was quantified by densitometric analyses. Non-treated EV cells were used as a control for the calculation of fold change. Data are presented as the mean ± SD (n≥3); **P*<0.05, ***P*<0.01.

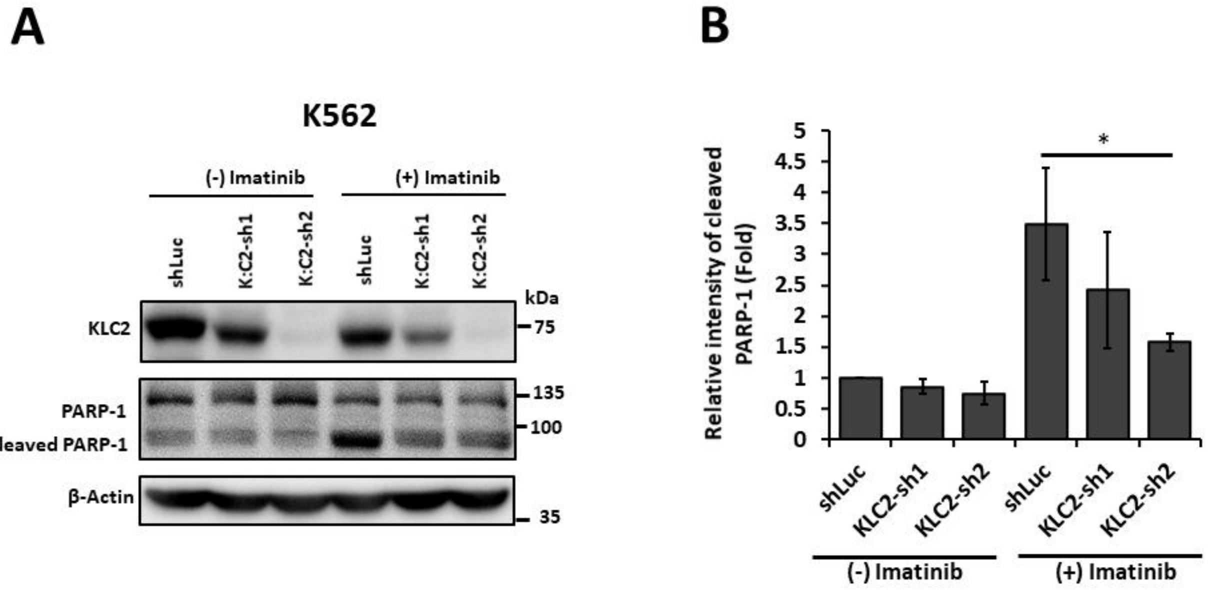

Figure S6

Figure S6. Knockdown of *KLC2* from CML cells decreased imatinib sensitivity with reduced apoptosis signals (related to Figure 2). (A) K562 cells stably knockdown of *KLC2* and treated without imatinib or with 400 nM imatinib for 72 h, indicated proteins were detected by immunoblot analyses. β-Actin was used as a control for equal loading. (B) Quantitated cleaved PARP-1 protein by densitometry analyses. Non-treated shLuc cells were used as a control for the calculation of fold change. Error bars represent the mean ± SD of three independent experiments; *$P<0.05$.

A

Group	(-) Imatinib		(+) Imatinib	
	Total sites	Tumor formed	Total sites	Tumor formed
EV	8	4/8	6	3/6
KLC2-WT	8	4/8	6	3/6
R312W	8	7/8	6	5/6
L523I	8	6/8	6	5/6

B

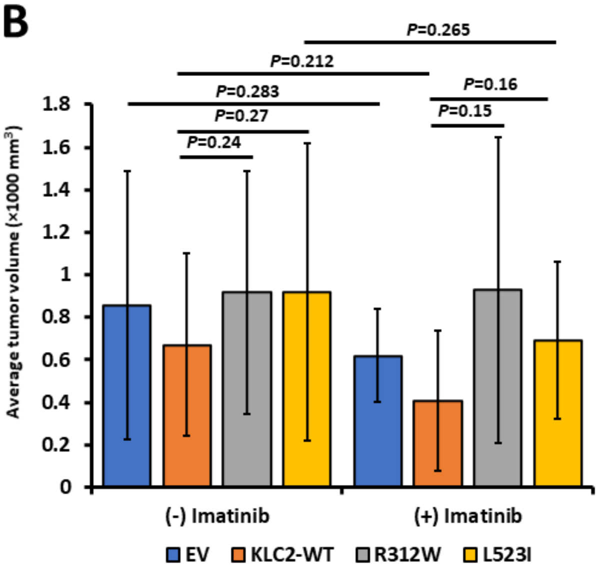

C

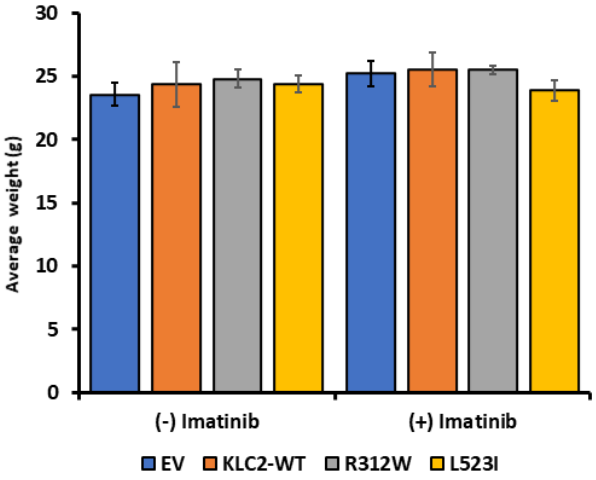

Figure S7

Figure S7. KLC2 mutants enhanced the tumorigenic activity and reduced the sensitivity of imatinib to CML cells in nude mice. KLC2-WT/MT and EV control stably transduced K562 cells inoculated in BALB/C nude mice. After a 10-day inoculation, the mice were randomized to the control group (receiving the vehicle alone) or the drug-treated group and administered for 15 days. Incidence of tumor formation with and without treatment of imatinib following KLC2-WT/MT and EV control K562 cells inoculation in mice (A), average tumor sizes (B), and average mouse body weight (C) were presented as mean ± SD.

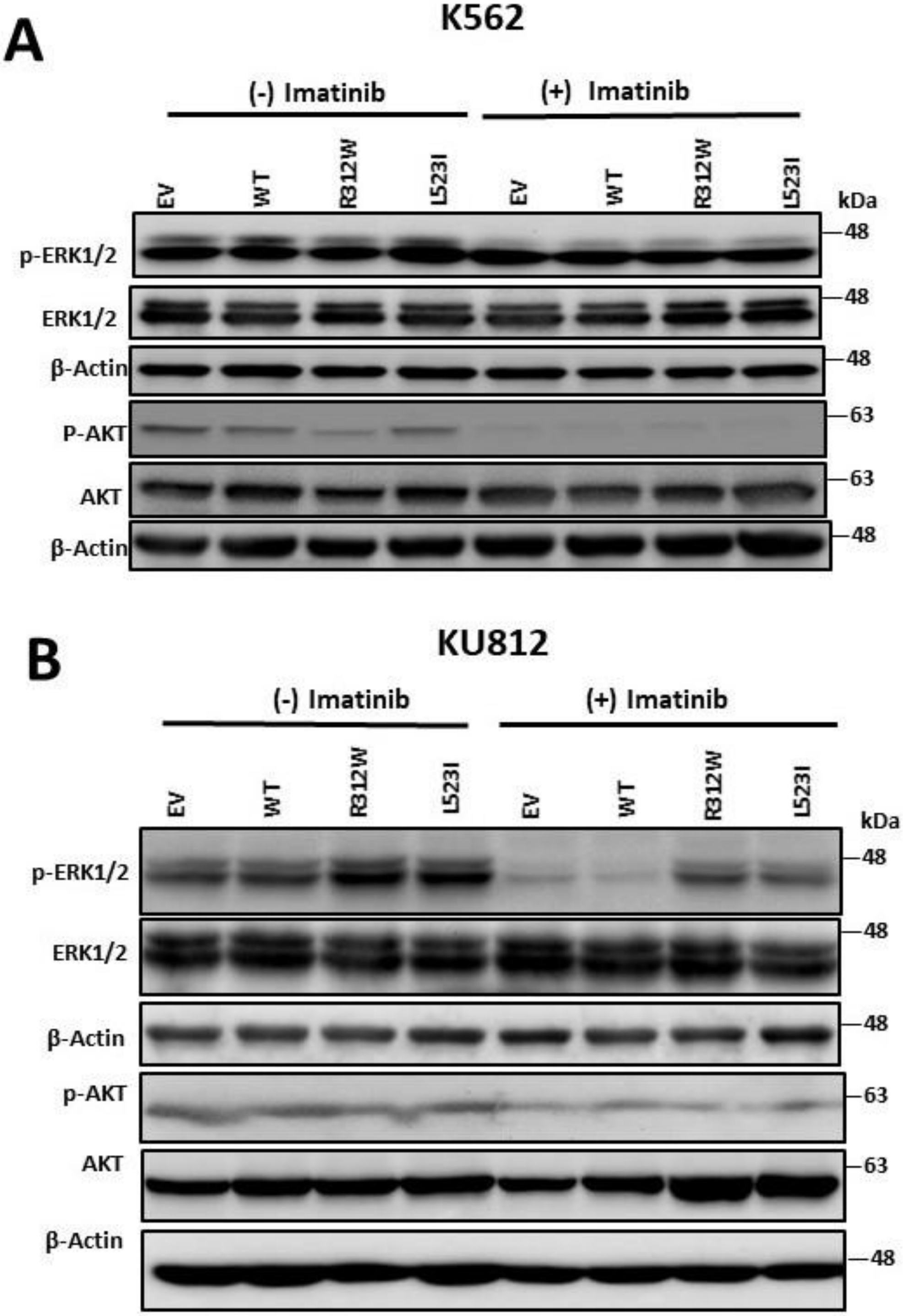

Figure S8

Figure S8. KLC2-mutant altered the cell growth signaling protein levels in imatinib-treated CML cells (related to Figure 3). (A–B) KLC2-WT and mutants-transduced stable K562 (A) and KU812 (B) cells were treated with 1 μM imatinib or without imatinib for 20 h and cell signaling proteins were detected with the indicated antibodies. β-Actin was used as a control for equal loading.

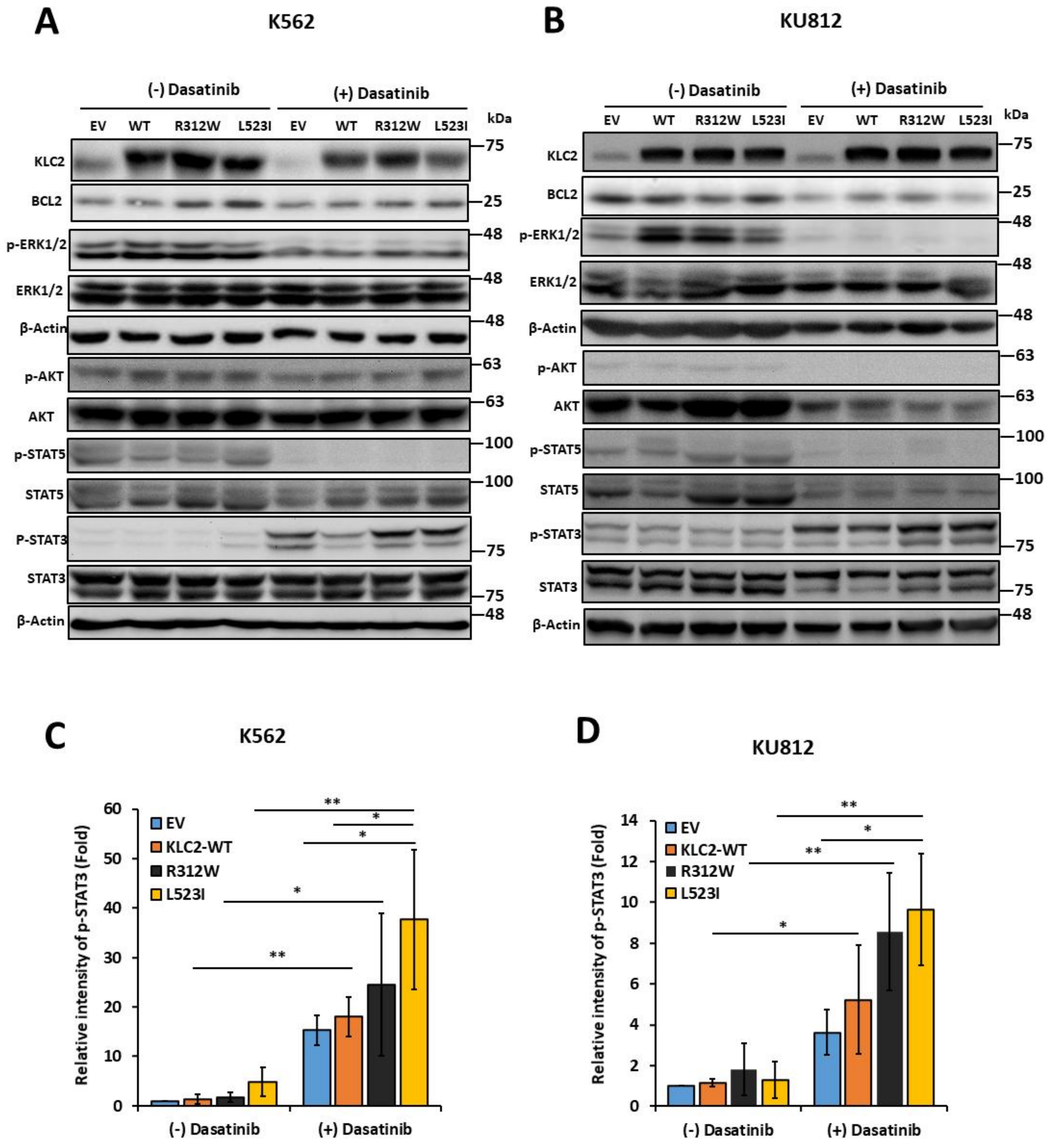
A
K562
(-) Dasatinib
(+) Dasatinib
EV
WT
R312W
L523I
kDa
KLC2
BCL2
p-ERK1/2
ERK1/2
β-Actin
p-AKT
AKT
p-STAT5
STAT5
P-STAT3
STAT3
75
25
48
63
100
B
KU812
C
K562
Relative intensity of p-STAT3 (Fold)
EV
KLC2-WT
R312W
L523I
D
KU812

Figure S9

Figure S9. KLC2-mutants increased STAT3 activation in dasatinib-treated CML cells (related to Figure 3). (A–B) KLC2-WT and mutants-transduced stable K562 (A) and KU812 (B) cells were treated with 1 nM dasatinib or without dasatinib for 20 h and cell signaling proteins were detected with the indicated antibodies. β-Actin was used as a control for equal loading. (C–D) Quantified pSTAT3 protein by densitometric analyses in K562 (C) and KU812 (D) cells. Non-treated EV cells were used as a control for the calculation of fold change. Error bars represent the mean ± SD of four independent experiments; *P<0.05, **P<0.01.

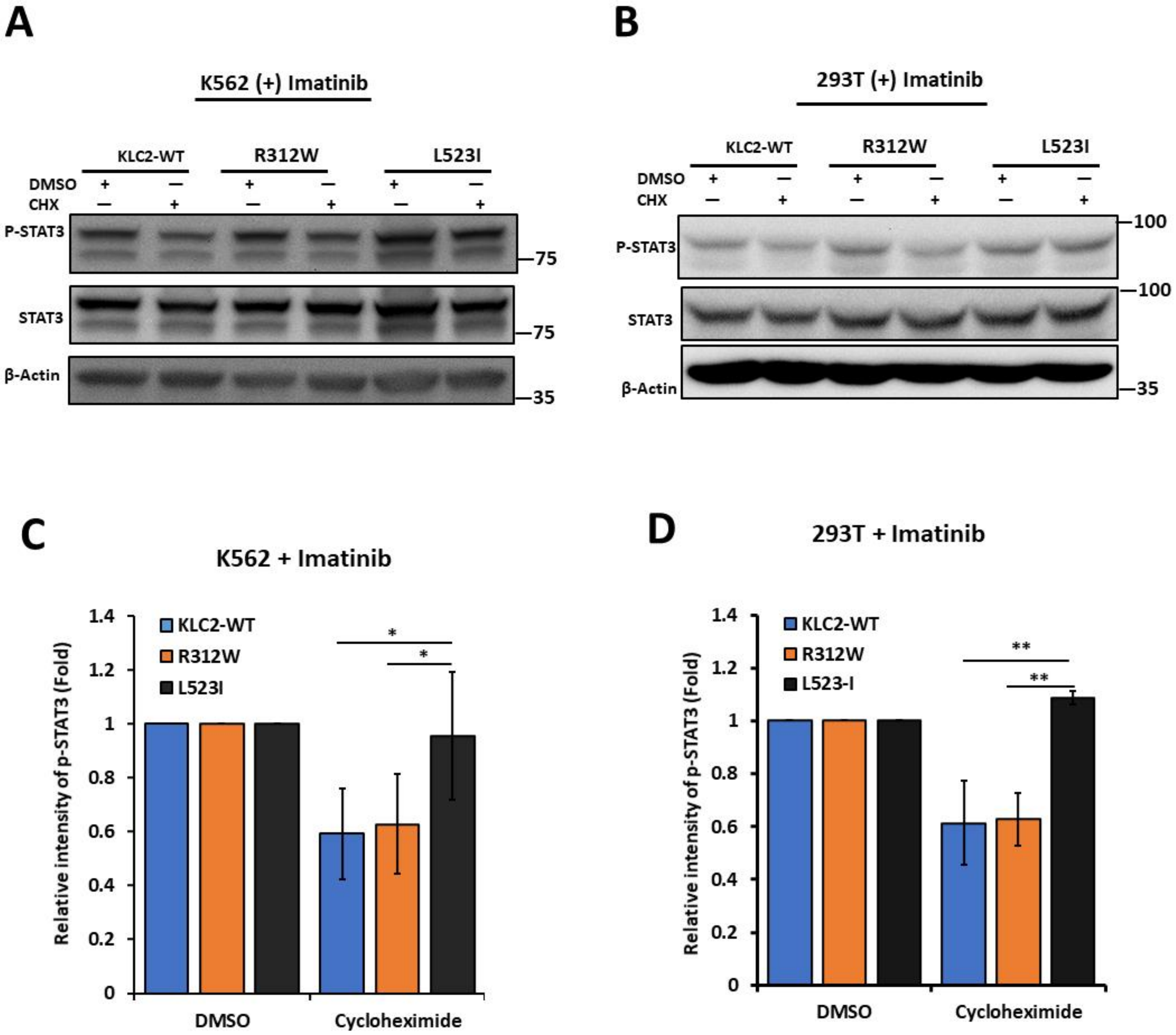
A
K562 (+) Imatinib
KLC2-WT
R312W
L523I
DMSO
CHX
P-STAT3
STAT3
β-Actin
75
75
35
B
293T (+) Imatinib
KLC2-WT
R312W
L523I
DMSO
CHX
P-STAT3
STAT3
β-Actin
100
100
35
C
K562 + Imatinib
Relative intensity of p-STAT3 (Fold)
KLC2-WT
R312W
L523I
DMSO
Cycloheximide
D
293T + Imatinib
Relative intensity of p-STAT3 (Fold)
KLC2-WT
R312W
L523-I
DMSO
Cycloheximide

Figure S10

Figure S10. Stability of pSTAT3 in KLC2-WT/mutants-expressing K562 and 293T cells with the treatment of imatinib (related to Figure 3). (A) 1 μM imatinib was treated in stably expressed KLC2-WT/MT K562 cells for 20 h then cycloheximide (CHX) (100 μg/mL) was treated for 6 h. Control cells were treated with DMSO only. Cell lysates were immunoblotted with antibodies against indicated proteins. (B) 10 μM imatinib-treated transiently expressed KLC2-WT/MT in HEK293T cells for 20 h then cycloheximide (CHX) (100 μg/mL) was treated for 6 h. Control cells were treated with DMSO only. Cell lysates were immunoblotted with antibodies against the indicated proteins. (C) Densitometry analyses of pSTAT3 expression in the cycloheximide and DMSO-treated cells. Error bars represent the mean ± SD of five independent experiments; *$P<0.05$. (D) Densitometry analyses of pSTAT3 expression in the cycloheximide and DMSO-treated cells. Error bars represent the mean ± SD of three independent experiments; **$P<0.01$. The values in the immunoblot data indicating relative signal density corresponding to β-Actin expression and immunoprecipitation were standardized relative to the treatment of DMSO. Representative data are shown.

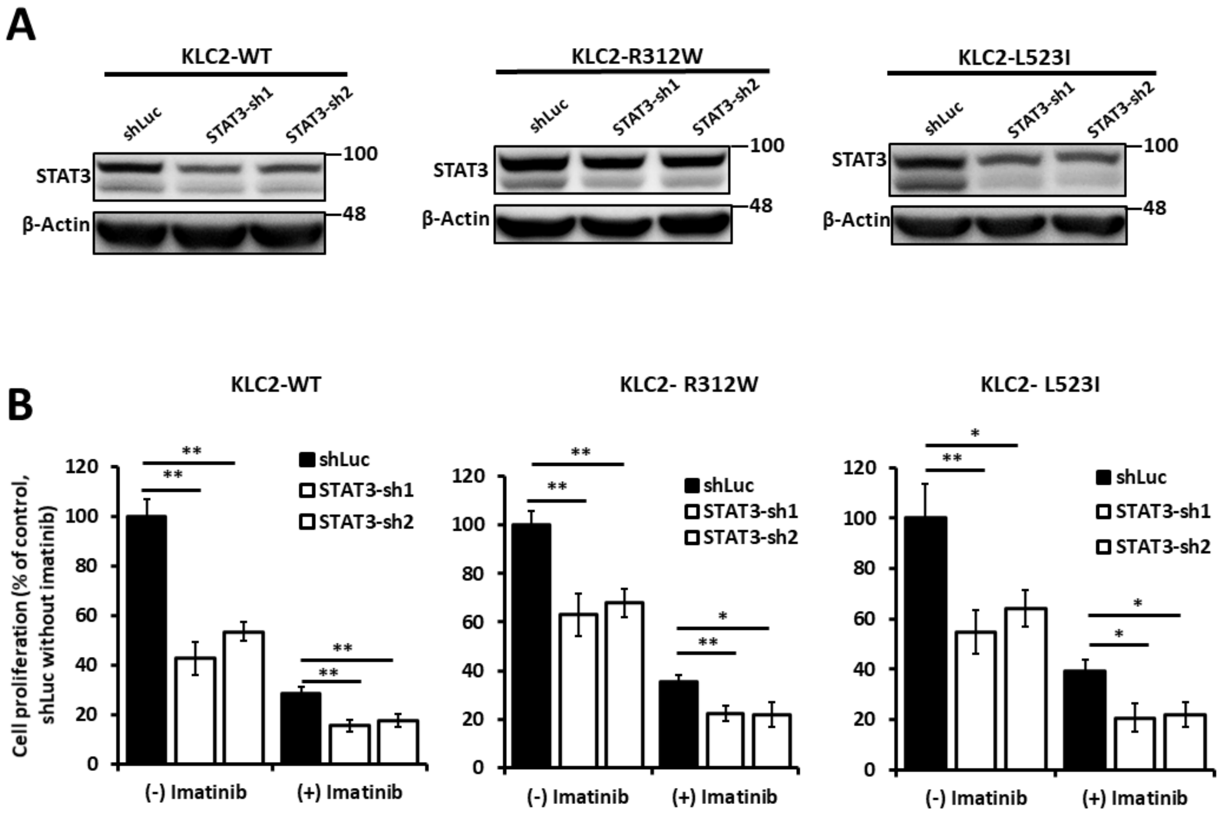

Figure S11

Figure S11. *STAT3* knockdown decreased the proliferation advantage and imatinib resistance to KLC2-WT/mutants- transduced K562 cells (related to Figure 3). (A) Knockdown of *STAT3* from KLC2-WT and mutant transduced K562 cells using two independent shRNAs and scrambled (shLuc), and silencing efficiency was checked by immunoblot analyses after 5 days of lentivirus infection. (B) Similar cells were assayed for cell survival with 400 nM imatinib or without imatinib for 72 h and viable cells were counted. Error bars represent the mean ± SD of biological triplicates; *P<0.05, **P<0.01.

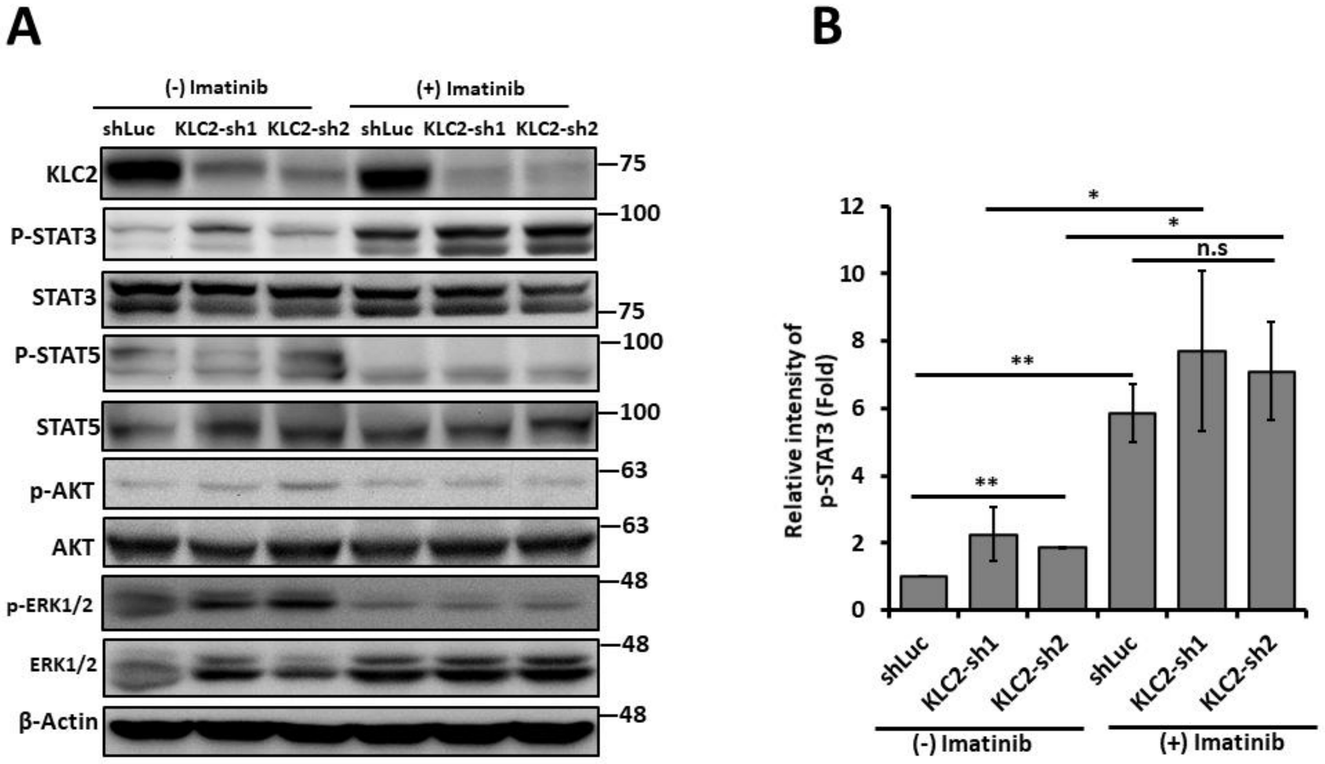

Figure S12

Figure S12. Knockdown of *KLC2* increased STAT3 activation in CML cells (related to Figure 3). (A) Knockdown of *KLC2* from K562 cells using two independent shRNAs and a scrambled (shLuc) lentivirus. Immunoblot data showing the silencing efficiency of KLC2 and indicated protein expression with 1 μM imatinib or without treatment for 20 h. β-Actin was used as a control for equal loading. (B) Quantified pSTAT3 protein by densitometric analyses. Non-treated shLuc cells were used as a control for the calculation of fold change. Error bars represent the mean ± SD of two independent experiments; **P*<0.05, ***P*<0.01. n.s, not significant.

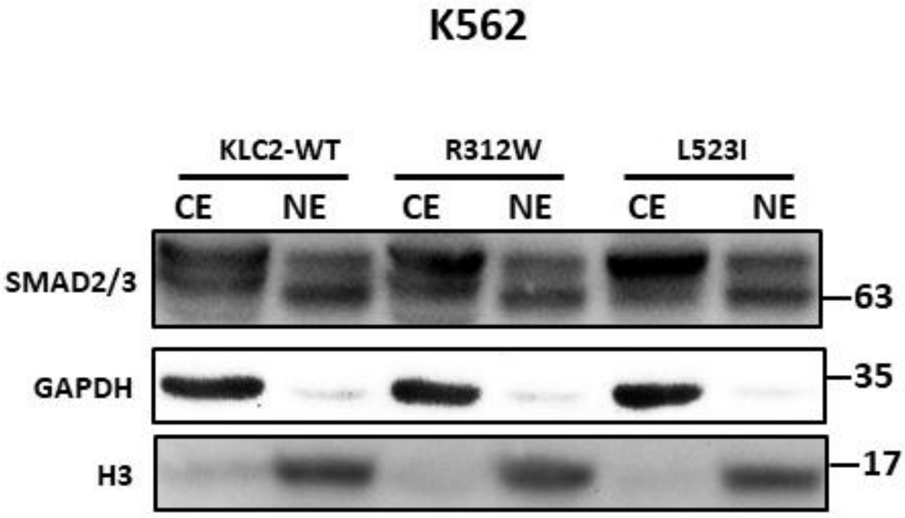

Figure S13

Figure S13. The cytoplasmic and nuclear distribution of SMAD2/3 protein in K562 cells (related to Figure 4). The cytoplasmic and nuclear distribution of SMAD2/3 protein was stepwise separated from stable K562 cells expressing KLC2-WT/MT analyses by Immunoblot using antibodies as indicated. CE indicates cytoplasmic protein extract, and NE, nuclear protein extract.
